# From isolating to blending contrasting self-attributes: a grounded theory of professional identity development in undergraduate architecture, engineering, and construction women

**DOI:** 10.3389/fpsyg.2026.1797683

**Published:** 2026-06-04

**Authors:** Andrea Nana Ofori-Boadu, Rabiatu Bonku

**Affiliations:** 1 Emerging Built Environment Women Center, Department of Built Environment, College of Science and Technology, North Carolina Agricultural and Technical State University, Greensboro, NC, United States; 2 Department of Industrial and Systems Engineering, College of Engineering, North Carolina Agricultural and Technical State University, Greensboro, NC, United States

**Keywords:** broadening participation, career development, female, higher education, identity development, pre-college education, qualitative methodology, STEM workforce development

## Abstract

**Introduction:**

Scholars call for more studies to better understand how women in masculine and male-dominated (MM) STEM professions construct professional identities, while navigating gendered tensions. Yet, studies on professional identity development (PID) processes in undergraduate architecture, engineering, and construction (AEC) women are sparse and mostly adopt structuralist perspectives and cross-sectional approaches which do not fully capture agency and trajectories in PID processes. The purpose is to construct a substantive theory that explains how self-attributes and engagements interact and progress as undergraduate AEC women construct their own professional identities.

**Methods:**

Adopting a constructivist and longitudinal grounded theory methodology, we utilize constant comparative analysis to analyze data from seven rounds of interviews with 78 undergraduate AEC women enrolled in five U.S. institutions.

**Results and discussion:**

We extend PID literature by providing a more nuanced and holistic understanding of agency and trajectory in AEC-PID as AEC women undergraduates progressively think, feel, and act as AEC women professionals. Our novel, *From Isolating to Blending Contrasting Self-Attributes (IBCS)* grounded theory explains how women gradually construct and integrate contrasting self-attributes to align with the contrasting attributes of AEC professions. Our new ‘*womfessionalization*’ concept captures a collectivist resilience mechanism by which STEM women with professional self-efficacy and motivation resolve gendered tensions by pursuing influential problem-solving and altruistic roles to transform innovation and inclusion for all within MM STEM contexts.

**Conclusion:**

Our IBCS framework links diverse identity content, process, and context dimensions and highlights the importance of considering agency and trajectories in STEM women’s PID dialogues, theory, research, and practice. We contribute to efforts to broaden women’s participation in MM STEM professions to diversify innovation and reduce persistent workforce shortages.

## 1 Introduction

Gendered tensions manifest as cognitive and emotional conflicts within women who encounter the systemic inequalities associated with being a woman and a professional in masculine and male-dominated (MM) STEM contexts (Wegemer and Eccles, 2019; Ofori-Boadu and Ofori-Boadu, 2022). Cheryan et al. (2017) emphasized that while women in STEM disciplines like biology are well-represented, women in engineering, physics, and computer science remain underrepresented in these MM STEM disciplines. To explain these larger gender gaps, Cheryan et al. (2017) emphasized: (a) MM cultures signal a lower sense of belonging to women; (b) women lack sufficient early experience, and (c) the presence of gender gaps in self-efficacy. Rather than attributing underrepresentation of women to lack of ability and interest, Cukier (2026) attributed to structural barriers to include chilly climates, misogynistic attitudes, microaggressions, and gender bias in pay and performance evaluations. Moss-Racusin et al. (2012) and Wise students (2022) noted that both male and female professors at a university rated female applicants as less competent than identical male applicants. Short (2019) and Trefts (2019) highlighted the imposter syndrome and explained that women work harder than required to prove that they earned their positions in STEM. Gendered tensions generate gender inauthenticity (Hatmaker, 2013), being invisible as engineers (Faulkner, 2009), amplification of gender over engineer (Hatmaker, 2013), physical surroundings (Du, 2006), and devaluation of altruism (Seron et al., 2018). Hatmaker (2013) stated that these tensions challenge how women negotiate and develop professional identities because they feel devalued as engineers.

To function and persist, women adopt identity management tactics to include impression management, confrontation, feminism, STEMinism, survival mentality, and gender re-direction (Ryan et al., 2020; Seron et al., 2018; Leaper and Arias, 2011; Powell et al., 2012; Ofori-Boadu and Ofori-Boadu, 2022; Saunders and Kashubeck-West, 2006). Powell et al. (2009) described how women undergraduates devalue femaleness to gain male acceptance. Yet, Seron et al. (2018) described them as muting marginalization criticisms and celebrating femininity; but, rejecting feminist identities because feminists’ complaints and demand for affirmation suggests that women cannot meet performance standards. Ruiz (2020) explained that while feminism represents advocacy of women’s rights based on equality, STEMinism focuses on increasing women’s representation in STEM contexts. Though, some tactics are unsuccessful and leave some women still feeling devalued, other tactics increase sense of belonging and PID (Hatmaker, 2013). Nevertheless, Betz (2023) and Fouad et al. (2023) cautioned against the generalization of findings as differentiations exist among women sub-groups. The American Association of University Women (2024) concurs because representation in STEM disciplines like biology has improved but underrepresentation persists in MM STEM disciplines like architecture, engineering, and construction (AEC). This suggests that broader STEM women theories may not be fully applicable in AEC contexts due to AEC’s listing among high-risk occupations (Stergiou-Kita et al., 2015) and non-traditional occupations for women (Lewis and Shan, 2024) which make AEC less attractive to women (Rotimi et al., 2022; Coskun et al., 2024).

The AEC industry develops built environments and employs about 8% of the global workforce (McKinsey, 2024). Significant growth is predicted (United States Bureau of Labor Statistics, 2025) and yet, 80% of U.S. firms face workforce shortages which may worsen due to challenges in attracting and developing workforce (Associated General Contractors of America, 2025). Its functioning is largely dependent on human talent, so workforce shortages limit its capacity to meet demands. Although increments in women’s participation could reduce workforce shortages, they are only 10.9% of the industry (McKissak, 2023). This undermines the full integration of diverse perspectives, creativity, and social relevance into the development of built environments (Volpe, 2024). Like other MM STEM women, gendered tensions in MM AEC contexts (Heydari et al., 2024) result from masculine nature, marginalization, discrimination, alienation, gender stereotypes and biases, and challenges in field and managerial roles. Women must work harder to earn respect and Arditi et al. (2013) found that men managers rated themselves as better than women managers in resilience and decision-making abilities, while women managers rated themselves as being more sensitive. To reduce these gendered tensions and improve underrepresentation, Heydari et al. (2024) recommended mentoring, role-modeling, education, outreach, and further research. On the personal level, Heydari et al. (2024) recommended deep psychological actions to strengthen women’s self-perceptions and counteract stereotypical beliefs about lacking the abilities and toughness needed for AEC professions.

Nevertheless, recruiting and retaining women in AEC professions remains challenging because these structural barriers and systemic gendered inequities are deeply engrained in AEC contexts. Concurring with Cheryan et al. (2017), Fash and Ofori-Boadu (2025), and United Nations Educational, Scientific and Cultural Organization (2017) noted that early socialization barriers deter girls’ interests through misconceptions and marginalization concerns. Although female and male performance in STEM are par, female students are less confident about their abilities and less willing to enroll in STEM courses (Ruiz, 2020). Reflecting industry trends, women also maintain low participation in undergraduate programs and were 14% of civil engineering (American Society of Civil Engineers, 2020) and 9% of construction (Lee, 2024) undergraduates. Women undergraduates also encounter gendered tensions and struggle with microaggressions (Sawruk, 2022) and socio-cultural (Adogbo et al., 2015) challenges. Ofori-Boadu et al. (2020) found that conflicts exist between woman and professional identities and reduce persistence. Ofori-Boadu and Ofori-Boadu (2022) noted that 95% of first-year women in her study encountered academic, psychological, and diversity tensions. Over time, these multi-level and multi-context tensions constrain attraction, professional identity development (PID), and persistence towards AEC and other MM STEM professions (Hernandez et al., 2017; Hatmaker, 2013; Sullivan and Kedrowicz, 2012). Yet, some women persist to become professionals. Empirical studies are needed to increase our understanding of PID in these women undergraduates who are in training to become woman professionals in MM STEM contexts (Cech, 2015; Ofori-Boadu and Ofori-Boadu, 2022; Ofori-Boadu et al., 2026).

Professional identity is self-representation that evolves in stages over time and through socializations by which the attributes of professions are aligned and integrated with self-attributes, resulting in an individual thinking, acting, and feeling like a professional (Cruess et al., 2014). Well-formed professional identities are critical for learning, well-being, and persistence from non-membership to professional membership (Burleson et al., 2021; Zhao, 2022; Hernandez et al., 2017). PID is understood through psychology and sociology frameworks which conceptualize identity as self-attributes that characterize an individual in a role, social group, or as a person (Burke, 2025; Gee, 2000; Swann and Bosson, 2008). Therefore, professional identity is the constellation of important self-attributes and meaningful engagements by which individuals define themselves and align with professional groups (Ibarra, 1999; Kunrath et al., 2020). Profession-relevant self-attributes include abilities and interests that contribute to an individual’s sense of being a professional (Burke, 2025). Profession-relevant engagements are meaningful socialization experiences that equip individuals with professional expertise and values (Nadelson et al., 2015; Trede et al., 2012).

Professional socialization experiences are largely shaped by professionals within an occupational context. Saks (2012) referred to professionals as knowledgeable experts with discipline-specific qualifications, as well as diagnostic and prescription powers. These powers are conferred through institutions that allow professionals to operate within designated boundaries. Professional boundaries are essential disciplinary, legal, ethical, and organizational limits which produce and sustain distinctions that are common to members and distinguish them from other professions (Farchi et al., 2023). Historically, specialized education was not needed for professional practice. However, with societal and technological advancements, formal education and/or licensure are now requirements. To become a member of a profession, Groen (2017) highlights undergraduate education contexts as formal and specialized learning sites. STEM undergraduates participate in academic and industrial STEM engagements that are developed through inputs from experts such as professors, practitioners, regulators, and accreditation agencies. They learn professional practice through direct and indirect engagements with professionals. By serving as instructors, role models, and mentors who maintain quality relationships in engagements, professionals (Crocetti et al., 2023) help undergraduates understand professional knowledge, norms, and values. Therefore, undergraduate STEM contexts are viable learning sites where professionals specify the professional attributes needed for undergraduates to transition from non-membership to membership. The meanings of professional engagements shape undergraduates’ specification of the attributes of professions and subsequent PID (McCall et al., 2021; Burleson et al., 2021). Therefore, professional socialization theories increase our understanding of how meanings from professional engagements influence STEM undergraduates’ specification of the attributes of professions to influence PID and commit to becoming professionals (Meeus et al., 2010).

To understand PID, some scholars utilize social-psychological approaches that highlight identity content (de Moor and Olaru, 2025) which consist of the self and professional attributes that align and integrate to drive PID within an individual towards a profession. Wagenschwanz (2021) conceptualized identity content as the meaning of an identity and identity structure as the hierarchical relationships among the different identities within an individual. Identity content studies draw from personal and social identity frameworks. Personal identity frameworks place emphasis on the attributes within self that make an individual unique and different from others (Stets and Burke, 2025). Personal identities are master identities which are salient in multiple situations and influence meanings associated with other self-attributes, engagements, and contexts (Stets and Burke, 2025). They are intertwined with personal agency and drive the determination of important self-attributes, meaningful engagements, and stable commitments. Therefore, personal identity theories increase our understanding of how unique self-attributes influence agency and identity content in STEM undergraduates’ professional identities (Meeus et al., 2010). On the other hand, social identity frameworks place emphasis on self-attributes that are similar to others and make individuals self-categorize as members of groups (Tonso, 2006). Tajfel and Turner (2004) highlighted how in-group members share meanings that influence coordinated behaviors to maintain group boundaries and restrain out-of-group individuals. To resolve discrepancies within group boundaries, individuals utilize strategies like social competition, social creativity, and individual mobility (Alparslan and Akdoğan, 2022; van Bezouw et al., 2021). Shuman et al. (2025) noted that disadvantaged groups often utilized social competition strategies to advance collective action and efforts for social change. In PID contexts, the social identity theory (Tajfel and Turner, 2004) increases our understanding of how meanings of professional socializations (McCain, 1985) with academic and industrial communities (Lave and Wenger, 1998) within disciplinary contexts cause undergraduates to adopt professional attributes. Therefore, PID involves the positioning of individuals by themselves and by professionals. Social identity theories increase our understanding of how similar self-attributes drive undergraduates’ PID through self-categorization (Meeus et al., 2010). Notably, individuals with similar self-attributes tend to be attracted to similar professions and individuals within similar professions tend to develop similar self-attributes (Rossetti et al., 2025).

Other established PID scholars utilize developmental approaches that highlight identity process dimensions over time as PID involves the iterative phases of exploration, commitment, and re-commitment to professions (Cruess et al., 2014; Jarvis-Selinger et al., 2012; Crocetti et al., 2023). Through intrapersonal and interpersonal processes within professional contexts, individuals specify self-attributes, explore the attributes of professions, and commit to preferred professions (Crocetti et al., 2023; Stets and Burke, 2025). Over time, individuals may encounter new experiences, explore the attributes of other professions, re-specify important self-attributes, and commit to other professions.

Considering that individuals desire to be both different and like others concurrently and in the same context, identity negotiation frameworks highlight interactions between personal and social identities that cause discrepancies within self (Ting-Toomey, 2017). Burke (2025) theorized that meanings made from significant engagements related to a specific identity are cognitively stored in an individual’s identity standard. This standard provides a reference point by which individuals negotiate new situational meanings in new engagements. A match between the standard and situational meanings results in identity verification (Burke, 2025). Crocetti et al. (2023) underscored reciprocal and transactional processes after self-verification when individuals develop commitments within specific contexts and express agency by actively shaping those contexts. Nevertheless, in situations where identity is not verified, discrepancies cause tension because the demands for social similarities infringe upon desires for personal differences (Branje et al., 2021). Kreiner et al. (2006) noted that an optimal balance is essential as overemphasizing differentiation causes disconnectedness, while overemphasizing similarities stifles creativity. To maintain distinctiveness, coherence, and continuity, individuals utilize various cognitive and behavioral mechanisms to revise identities so they can resolve these tensions and maintain identity congruence (Ting-Toomey, 2017). Agency frameworks elucidate how individuals intentionally influence their own functioning through iterative exploring, understanding, assessing, specifying, negotiating, and integrating personal and professional identities (Bandura, 2001; Eteläpelto et al., 2014; Chávez et al., 2023; Sawatsky et al., 2023). However, undergraduates can either feel empowered or constrained to enact agency (McIntyre et al., 2020). Also, agency can vary by individual differences, intensity, time, situation, context, nature, direction, options, and available resources in a specific context. Eteläpelto et al. (2014) emphasized that agency fosters the maintenance or re-negotiation of professional identities to include pursuing creative initiatives, crossing professional boundaries, and transforming work practices. Rather than selecting a social-psychological or developmental approach in understanding identity development, Crocetti et al. (2023) recommended the cross-fertilization approach to integrate these approaches. Also, Stets and Burke (2025) advocated for theories that explain how the meanings of identities originate and evolve over time within specific contexts, particularly those with marginalized populations like MM STEM women who develop professional identities while while navigating gendered tensions.

Gendered tensions involve conflicts that originate from expectations and norms that restrict women to traditional feminine roles and restrain them from advancing into roles that are perceived as masculine. It is well-documented that women experience gendered tensions in MM STEM professional contexts because of conflicts between traditional feminine attributes (e.g., communal, altruistic) ascribed to women and stereotypical masculine attributes (e.g., agentic, competitive) ascribed to STEM professions (Eagly and Karau, 2002; Hatmaker, 2013). Seron et al. (2018) noted that educational contexts successfully reproduce the working culture of related professions through socialization processes and so mimic the gender biases of the workplace. Through engagements such as initiation rituals and industrial experiences, men undergraduates are encouraged to solve practical and technological challenges while women are relegated to nontechnical and social roles which are denigrated in these contexts and so undermine women’s confidence (Seron et al., 2016). Established scholars have investigated gendered tensions by drawing from the role congruity theory which holds that individuals in a social group will be negatively evaluated when the characteristics stereotypically associated with their gender are incongruent with their role (Eagly and Karau, 2002). Therefore, women are expected to be a better fit for feminine roles in nurturing and social professions like nursing, and a weaker fit for the masculine agentic roles in STEM professions (Faulkner, 2009; Yang and Barth, 2015). These stereotyped beliefs result in women being perceived as lacking the interests and abilities needed for MM STEM professions. Bloodhart et al. (2020) noted that women excel in STEM but are still subject to bias even if they outperform male counterparts. The role congruity theory suggests that women are not interested in STEM professions because they do not accommodate traditional feminine goals (Yang and Barth, 2015; Diekman and Eagly, 2008). Therefore, cognition, behavior, PID, and persistence are significantly constrained in MM STEM women because beliefs limit them to traditional feminine roles (Baguant, 2021). Consequently, some women self-withdraw from engagements and contexts that present gendered tensions such as those lacking numerical women representation and this spurs subsequent underrepresentation (Ryan et al., 2020).

To improve women participation in STEM contexts, the above-mentioned frameworks have been utilized to understand PID. Yet, existing AEC women studies are few and mostly align with structuralist underpinnings that emphasize systemic gender inequalities and structural controls that position women having limited control over PID. Women are positioned as heavily reliant on structural supports such as family, mentors, women faculty, and women organizations (Adogbo et al., 2015; Sawruk, 2022; Baguant, 2021; Shane et al., 2012). They are conceptualized as passive followers who are in a homogenous group that primarily responds in the same way to threats (Makhathini and Aigbavboa, 2025) and are restricted to systems that facilitate gender tensions and constrain options (Gao, 2007; Vijayaragunathan and Rasanthi, 2019). While women have similarities in identity threats and PID, it is also unrealistic to ignore individual differences and identity opportunities (Bataille and Vough, 2020). Existing studies have increased our understanding of systemic barriers and structural supports, but little is known about the diverse and agentic negotiation mechanisms utilized by women to subjectively resolve gendered tensions in PID. Little attention is given to how diverse self-meanings and subjective interpretations of meaningful engagements cause variations in PID. To gain a more nuanced understanding of PID and inform the development of tailored interventions, constructivist underpinnings will better highlight individual differences and agency in PID (Makhathini and Aigbavboa, 2025; Gao, 2007; Ford et al., 2021). Yet, limited studies adopt constructivist perspectives.

Furthermore, the few existing constructivist studies mostly utilize cross-sectional designs that provide single-point data and lack dimensions connected by time or process. In their study of first-year women, Ofori-Boadu and Ofori-Boadu (2022) highlighted how AEC-PID was driven by agency and self-attributes like being creative, hands-on, and female. Smith et al. (2020) concurred and found an association between creativity and PID in women who participated in a one-day conference. In her dissertation on how internships shaped PID, Armstrong (2023) found that AEC women of color undergraduates increased in awareness of salient identities, agency, and sense of direction to move forward. Ofori-Boadu et al. (2026) reported interactions between realistic creativity self-attributes and PID processes in AEC women undergraduates. While these studies contribute to understanding agency in PID in women undergraduates, like other cross-sectional design studies, they are limited because they do not fully capture nor trace real-time linkages across important self-attributes (Nadelson et al., 2015) and engagements that evolve across life stages as women construct their professional identities (Burleson et al., 2021). Considering that neither agency nor structure is static, and girls begin career reflections during pre-college (Ofori-Boadu et al., 2020), longitudinal studies will better capture the origination and varied trajectories of PID (Burleson et al., 2021). Yet, no longitudinal study explains the varied trajectories in AEC women’s PID.

With its constructivist perspectives, pseudo-longitudinal design, and undergraduate AEC contexts, McCall’s professional identity negotiation theory (McCall et al., 2021) could be useful in understanding AEC women undergraduates PID as it explains how civil engineering undergraduates integrate definitions of self and professions. However, it presents three limitations: (1) Majority of participants are men and so it offers limited understanding of women; (2) Due to its pseudo-longitudinal design, it does not realistically trace how identity content dimensions originate and evolve to influence PID; and (3) Due to its single-discipline and single-institution context, its generalizability into broader STEM contexts is limited.

To address these gaps, the purpose is to construct a substantive theory that explains how self-attributes and engagements interact and progress as undergraduate AEC women construct their own professional identities, while navigating gendered tensions. We integrate constructivist (Gao, 2007), developmental social-psychological (Crocetti et al., 2023), and role congruity frameworks (Eagly and Karau, 2002) to investigate agency and trajectories in AEC-PID. We expand PID literature by constructing the first grounded theory that provides a more nuanced and holistic understanding of how AEC women undergraduates construct professional identities, while navigating gendered tensions in AEC contexts. We hypothesize that women who possess AEC-relevant self-attributes that are important to them and participate in meaningful AEC-relevant engagements are likely to evolve over time and commit to becoming professionals (Hatmaker, 2013). Our constructivist underpinnings and longitudinal design allow us to trace specific identity content, process, and context dimensions that are important to women as they serve as their own agents in AEC-PID. We expect that there will be both similarities and differences in AEC-PID processes. Secondly, we provide a novel and useful model that integrates established identity and gender theories to inform tailored and phased investigations and interventions that advance PID in women undergraduates in MM STEM contexts.

We conceptualize AEC-PID as a dynamic process by which women undergraduates through meaningful engagements iteratively explore, make meanings, specify, construct, and integrate important self attributes to align with the attributes of AEC professions and commit to becoming women professionals (Burleson et al., 2021; Dryburgh, 1999). Our research questions are: (1) How do self-attributes and engagements interact and evolve to influence women’s decisions to enroll in undergraduate AEC programs? (2) How do self-attributes and engagements interact and evolve to influence how undergraduate women construct their own sense of becoming AEC professionals? We conceptualize self-attributes as personal and social self-meanings that evolve over time to influence AEC-PID (Groen, 2017). We conceptualize engagements as significant interactions within AEC contexts that influence undergraduate women’s thinking, feeling, and acting as professionals (Xu et al., 2023; Cruess et al., 2014). Commitments are conceptualized as enduring choices made to persist into becoming an AEC woman professional (Crocetti et al., 2023). We expect that while women with well-developed AEC professional identities commit and remain within AEC professional boundaries, women with constrained identities will explore other aspirations.

## 2 Methods

Our institutional review board approved study utilized a longitudinal and constructivist grounded theory (GT) methodology to increase our understanding of trajectories and agency in AEC-PID (Charmaz, 2014). Considering our focus on pre-college, university, and early-employment contexts, GT allows theory generation in an unbounded manner and deepens theoretical connections across contexts and time (McCall and Edwards, 2021). GT is utilized when a theory is unavailable to explain a social process (Xia et al., 2025). It involves simultaneous data collection, analysis, and interpretation for theory construction (Alemu et al., 2015). Our constructivist underpinnings permits the co-construction of a GT on the AEC-PID phenomenon by drawing from the varied meanings and experiential views of research participants (RPs) and researchers (Charmaz, 2014). Our longitudinal approach permits a four-year (2019–2023) investigation to understand how AEC-PID is constructed over time (Burleson et al., 2021).

### 2.1 Sensitizing concepts

Sensitizing concepts inform initial ideation and research design but do not force coding frameworks on data (McCall and Edwards, 2021). Social-psychological and developmental frameworks inform our exploration and tracking of identity content, process, and context dimensions that interact and evolve to contribute to AEC-PID (Crocetti et al., 2023; Stets and Burke, 2025; Ofori-Boadu and Ofori-Boadu, 2022). McCall et al. (2021) and Ofori-Boadu and Ofori-Boadu (2022) justify our conceptualization of undergraduate AEC contexts as viable learning sites for AEC-PID. Our longitudinal approach (Burleson et al., 2021) permits us to study the varied and evolving engagements that inform women’s iterative specifications of evolving professional and self attributes. Hence, we conducted seven rounds of interviews to understand how women construct identities over time and across contexts. Rather than a single discipline approach, we acknowledged similarities across AEC disciplines and adopted a multi-disciplinary approach (Ikudayisi et al., 2023). Drawing from person-environment fit theories (Holland, 1997; De Cooman and Vleugels, 2022), we hypothesized that, regardless of AEC disciplinary and individual differences, RPs are likely to have some similarities in AEC-PID. Therefore, RPs comprised of AEC women from five U.S. institutions. Informed by role congruity theory (Eagly and Karau, 2002), we acknowledge that the mismatch between woman and professional identities can cause gendered tensions and discrepancies in PID. Therefore, we conscientiously craft interview questions to gain insights into how women negotiate and resolve discrepancies. Gender socialization theories justify our classification of AEC environments as gendered socialization grounds where women engage differently from men and women identities are threatened (West and Zimmerman, 1987; Stergiou-Kita et al., 2015; Ofori-Boadu and Ofori-Boadu, 2022). Yet, with our constructivist perspectives, we remained open to diversity in how women undergraduates navigate identity opportunities (Bataille and Vough, 2020; Gao, 2007). Our narrative identity approach allows us to capture meaningful experiences shared by RPs through autobiographical reasoning and conversations during interviews (van Doeselaar et al., 2020).

### 2.2 Recruitment and enrollment

Faculty recruiters initially hand-distributed recruitment documents in first-year AEC courses to students who self-identified as women and were enrolled in undergraduate AEC programs in five U.S. institutions. Subsequent digital distributions to university communities had links for self-nominations. For geographic diversity, institutions were in the South, Midwest, and West U.S. regions. To strengthen robustness of findings, we utilized maximum variation purposeful sampling to enroll RPs (*N* = 78) with varied characteristics and experiences (Elliott and Lazenbatt, 2005). Enrollment from one Hispanic-serving (*N* = 7), two predominantly white (*N* = 33), and two historically black universities (*N* = 38) facilitated institutional and racial diversity. Institutions provided diverse offerings (e.g., minors) for multidisciplinary AEC education.

### 2.3 Data collection

We utilized 60-to-90-min semi-structured interviews which provided an initial structure and allowed follow up questions. Our critical incident interviewing technique elicited details about incidents and went beyond surface conversations to understand both individualized and shared meanings (Alemu et al., 2015). Questions focused on the *whats, whys, hows,* and *whens* associated with how meaningful engagements and important self-attributes interacted and evolved to influence shifts in thoughts, feelings, actions, and aspirations towards becoming a professional. Interview notes preserved context, tracked linkages, and informed subsequent questions. Over the four-year period, 78 RPs from architecture (*N* = 19), engineering (*N* = 39), construction (*N* = 14), and dual architecture and engineering (*N* = 6) programs participated in 190 interviews over seven rounds. Responses to questions in the first-round interviews (*N* = 40) were foundational to this study.

A round 1 question was “*Describe memorable experiences that you have of yourself as a girl and teenager*.” Responses reveal links between meaningful pre-college engagements and important gendered and non-gendered self-attributes. Another question was “*Describe any memorable pre-college experiences that contributed to your enrollment in the AEC program at your current institution*.” Responses reveal meaningful pre-college engagements that contributed to the origination of AEC-PID. Other questions were “*Describe memorable experiences that contributed to your AEC views, actions, competencies, performance, and recognitions as a girl and teenager*…. *Describe any memorable experiences that have occurred this semester to contribute to your desire to become an AEC professional… Describe key actions that you have taken towards becoming an AEC professional*.” To understand agency and trajectory, the responses reveal the meaningful undergraduate engagements that caused early shifts in thoughts, feelings, and actions towards becoming an AEC professional. Subsequently, two rounds of interviews were conducted each year during the first 3 years to monitor changes in AEC-PID. Questions asked included “*Describe an ideal female AEC professional. What influences your descriptions? How do* you compare with the ideal female AEC professional?… *Explain experiences, reasonings, and feelings that have strengthened your sense of becoming a female AEC professional …Explain experiences, reasonings, and feelings that have weakened your sense of becoming a female AEC professional… Explain strategies that you plan to implement to strengthen your sense of becoming a professional AEC woman… Some undergraduate AEC women associate their sense of becoming female AEC Professionals with their transitions from being AEC beneficiaries (recipients) to becoming AEC benefactresses (contributors). Using your own personal experiences, can you explain why you agree or disagree to this statement?* … The last question was one of the questions that allowed RPs to provide feedback on the emerging theme that the shift from beneficiary to benefactress mindsets reflected PID. The seventh-round interview captured maturation and professional commitments. Questions included, “*On a scale of 1 (not enthusiastic) to 5 (extremely enthusiastic), please rank how enthusiastic you are to become a professional AEC woman. Provide a rationale for your ranking…Explain strategies that you plan to implement to strengthen your sense of becoming a professional AEC woman*.”

An average of 27 RPs participated in each round and feedback on emerging themes was obtained during rounds 3–7. Six percent of RPs participated in all rounds and 14% participated in five or more rounds. The race distribution for the 14% were White (*N* = 5); African-American (*N* = 3), Asian (*N* = 2), and White-Hispanic (*N* = 1). Attrition and skipping of interviews were attributed to busy schedules, loss of interest, early graduation or major switches. Nevertheless, through continuous purposeful sampling, we enrolled additional RPs to validate emerging themes and increase the robustness of our emerging theory.

### 2.4 Data analysis

We utilized a bottom-up, inductive, and interpretivist approach to systematically understand patterns and trends (McCall et al., 2021). Interview transcripts were uploaded into the NVivo 12 software for constant comparative analysis that detected analytical distinctions by comparing statements across RPs, disciplines, engagements, time, and contexts. Our three coding stages involved open/focused, axial, and selective coding (McCall et al., 2021). During initial open coding, we noted the changes in self-attributes, engagements, strategies, and outcomes associated with changes in professional aspirations. Process coding facilitated the tracking of time dynamics to include emergence, sequences, and transitions (Saldana, 2011). Coding captured what RPs were “saying” as well as “doing with what they were saying” as they expressed meanings, thoughts, feelings, actions, and intentions related to constructing AEC-PID intentionally or unintentionally (McCall et al., 2021; Cruess et al., 2014). The first transcript yielded 32 open codes. Subsequent coding was increasingly abstract and revealed the properties and dimensions of emerging concepts. Focused coding ensured that the most frequent and relevant open codes were synthesized to explain larger segments of data. Focused codes that captured similar identity content and processes within RPs, as well as across RPs, AEC disciplines, AEC institutions, contexts, and time were clustered (Zunker and Ivankova, 2011).

With new meaningful engagements, RPs were found to iteratively specify, negotiate, construct, and re-specify multiple contrasting self-attributes within themselves to align with AEC professions. These self-attributes were clustered into categories and characterized as contrasting because RPs utilized terms such as “but,” “different,” “between,” and “opposite” to express how they perceived these self-attributes as contrasting. For example, creative (e.g., artistic) and analytical (e.g., mathematical) self-attributes were characterized as contrasting because the arts draw heavily on divergent thinking and subjective inspiration, whereas mathematics draws heavily on convergent thinking and objective reasoning (Razumnikova, 2013; Pallister, 2022). The contrasting self-attributes were further clustered into three focused codes (interest, ability, and resilience) which gained analytical relevance. The interest focused codes highlighted self-attributes (e.g., hands-on) that RPs expressed as providing enjoyment and the desire or motivation to re-engage with processes, spaces, and objects related to AEC aspirations (Hidi and Renninger, 2006). RPs utilized words such as “like” to align these interests with aspirations. The ability focused codes highlighted self-attributes (e.g., intelligent) that RPs expressed as providing the self-efficacy and confidence needed to succeed in AEC professions (Campbell, 2021). RPs utilized words such as “can” to align abilities with aspirations. The resilience focused codes highlighted self-attributes (e.g., toughness) that RPs expressed as providing the inner strength for adaptive functioning that facilitated sense of belonging as RPs resolved gendered tensions (Park et al., 2021). RPs utilized words like “tough skin,” “speak up” and “don’t have to change”. With increasingly complex, realistic, and gendered engagements, RPs constructed and re-specified self-attributes to align with the attributes of AEC professions. Therefore, categories related to significant shifts in engagements, self-attributes, strategies, and aspirations were clustered into a unifying phase. This yielded four sequential phases that captured RPs’ shifting aspirations to include non-AEC professional aspirations in phase 1, broad AEC professional aspirations in phase 2, specific AEC professional aspirations in phase 3, and specific AEC specialization aspirations in phase 4. During axial coding, we structured the categories within each phase to capture how categories related to each other. Therefore, each category within each phase was assigned to one of six GT components—context, cause, phenomenon, strategy, intervening condition, and outcome (Zunker and Ivankova, 2011). Table 1 provides the definitions for GT components and captures how key categories in phase 4 were structured to explain the AEC-PID phenomenon (McCall et al., 2021). The outcome category for each antecedent phase became the context category for the subsequent phase, establishing continuous links across phases to allow traceability of dimensions that explain AEC-PID.

**Table 1:** Grounded theory components of phase 4 of the IBCS grounded theory.

GT components	Definition	Isolating to blending contrasts grounded theory components
Context	The environment in which the core phenomenon occurs	Undergraduate AEC education.
Core phenomenon	The central occurrence or process which is being investigated	Becoming an AEC woman professional.
Causal conditions	The cause or source of the core phenomenon	Navigating new AEC expert engagements with identity affirming and conflicting situations. Specifying and re-specifying the contrasting attributes (*Professional Abilities; Realistic Interests; Transformational Resilience*) of the Blending Contrasting Self-attributes (BCS) professional identity standard.
Intervening conditions	Situations that influence strategies	Assessing and re-assessing person-BCS standard similarities and differences.
Strategies	Responses or purposeful actions that shape the core phenomenon	Negotiating, enacting, re-specifying, and integrating personal and professional self-attributes.
Outcome	Consequences or end results from the implementation of strategies	Committing to AEC professional aspirations (Boundary-conforming; Boundary-altering; and Boundary-breaking).

We utilized selective coding to refine the emerging theory by linking theoretical categories around the core category, *Becoming a Professional AEC Woman*, in the fourth phase. This core category captured codes related to RPs’ thinking, feeling, and acting like AEC women professionals. It was abstract enough to explain AEC-PID and relate to all categories. RPs experienced varied levels of discrepancies, and these caused the revision of some codes and categories to accommodate diversity. Extremely negative cases involved RPs who had switched to non-AEC programs. Despite their exit from AEC programs, three RPs were still interviewed in subsequent rounds to test, revise, and improve the robustness of the emerging theory. Three trajectories captured the sequence and clusters of varied mechanisms that RPs utilized to resolve discrepancies and influence aspirations. Theoretical sampling was conducted until saturation was achieved and all RPs in interview rounds 3 to 7 were categorized into one of these three trajectories (Alemu et al., 2015). With no new codes emerging, the model was finalized and theoretical saturation was achieved (Xia et al., 2025).

### 2.5 Quality

We comply with the four GT quality criteria of credibility, originality, resonance, and usefulness (Charmaz and Thornberg, 2021). For credibility, data was accessible and 13 assistants developed codes and memos to enhance diversity in analytical perspectives during team discussions. Memo-ing and coding was initially conducted independently with team members writing memos and coding the same transcripts; and, then collaboratively during team meetings where inconsistencies were resolved and agreement was reached (Delve, 2025). For originality, this is the first constructivist and longitudinal theory that provides a nuanced and holistic understanding of AEC-PID processes in women undergraduates.

To guarantee resonance, emerging themes were included in subsequent interviews to allow the co-construction of the emerging theory by both RPs and researchers. Literature reviews and feedback from GT scholars provided meaningful feedback to strengthen the emerging grounded theory. For usefulness, the theory informs investigations and interventions to support PID in MM STEM women.

### 2.6 Positionality

The first author is an AEC educator and researcher. Her relativist ontology and interpretivist epistemology influenced her adoption of the constructivist GT methodology. She leveraged her AEC expertise and experience in conceptualizing and implementing this study. Due to her role as an AEC woman professor, she had observed varied behaviors of undergraduate women. Therefore, she was eager to adopt a constructivist lens and utilize a longitudinal approach to investigate the varied and iterative internal mechanisms utilized to resolve discrepancies and construct identities, while negotiating gendered tensions. The second author is a woman with a doctoral industrial engineering degree and holds an academic administration position. Her non-AEC background brought diversity and rigor to the analytical process to ensure that theoretical findings were substantiated by data.

## 3 Results

**Figure 1:**
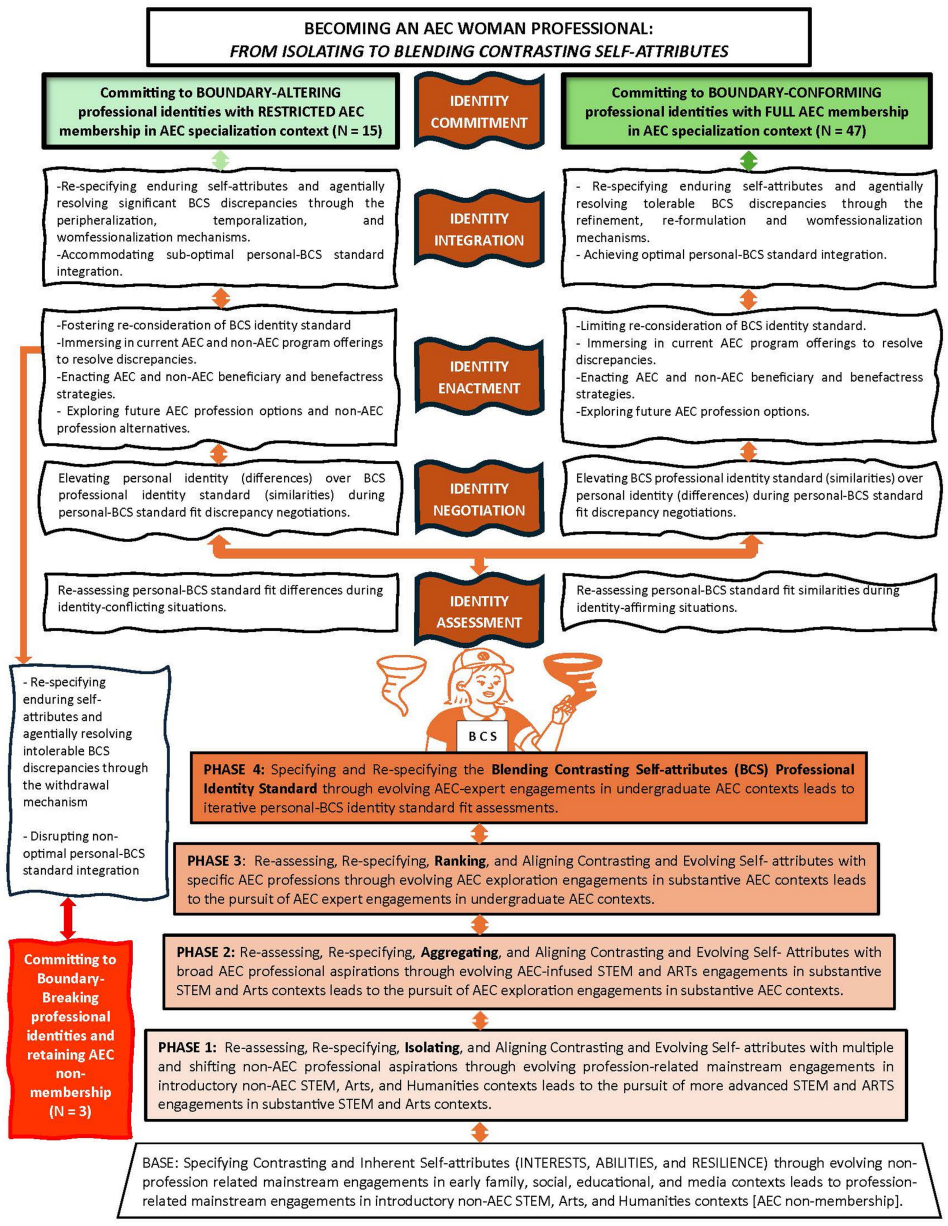
The “*From Isolating to Blending Contrasting Self-Attributes (IBCS)*” GT model.

We provide a more nuanced and holistic understanding of agency and trajectories in PID processes as AEC women undergraduates commit to becoming AEC women professionals, while navigating gendered tensions. Our, *From Isolating to Blending contrasting Self-Attributes (IBCS)* theory explains how women transition from isolating to blending contrasting self-attributes as they agentially construct and integrate their contrasting personal and professional attributes through increasingly complex, realistic, and gendered AEC engagements. This theory captures the progressive specification of professional and self attributes as RPs make meanings from new situations during engagements. A visual representation of our IBCS framework showing the key categories and focused codes within four sequential phases of the nuanced and holistic AEC-PID process are shown in Figure 1.

As shown in the white trapezoid at the base of Figure 1, interactions between contrasting non-professional mainstream engagements in early family and educational contexts and inherent contrasting self-attributes within RPs provided the basic requirements for the origination of professional aspirations. This is because RPs gain awareness and understanding of their own contrasting self-attributes through socialization. These self-understandings direct subsequent person-profession fit assessments and negotiations to inform professional aspirations and the pursuit of subsequent engagements in relevant contexts. Through interactions with subsequent engagements (causal conditions) situated in corresponding environments (context) within each phase, RPs conduct iterative person-profession assessments (intervening conditions) which reveal person-profession fit discrepancies. To resolve discrepancies, RPs conduct internal negotiations, enact strategies, re-specify, and integrate self-attributes (strategies) to align with the attributes of professional aspirations. These processes then drove RPs pursuit of subsequent and more advanced engagements within corresponding environments and contexts (outcome) to advance professional aspirations.

We utilize four loosely linked sequential phases to cluster the unique contexts, engagements, self-attributes, strategies, and aspirations for each phase as women progressively transition from non-membership (Phase 1) to AEC membership (Phase 4). These phases capture short-term progressions towards AEC-PID as shown by the four lower and increasingly darker, orange-shaded rectangles. Though there are variations in the contrasting self-attributes expressed by RPs for each of the four phases, these self-attributes mostly fell within the three broad dimensions of interests, abilities, and resilience and were traceable across phases. These three dimensions are captured with uppercase letters in the white trapezoidal base in Figure 1. Meanings from new engagements facilitate the re-specification of the self-attributes in these three dimensions and they gradually mature across the four linked phases and contexts. Therefore, the outcome categories of each phase became the context categories for the subsequent phase. Also, the unique and progressive strategies utilized by RPs to negotiate and construct self-attributes for each phase are bolded in the orange shaded rectangle for each phase. We utilize deeper shades of orange to move from lower to upper phases and this captures increasingly complex, realistic, and gendered engagements and contexts.

We utilize double headed arrows in between shaded rectangles to capture the iterative nature of this process. By captioning our theory as *From Isolating to Blending Contrasting Self-Attributes*, we capture the range of unique and progressive strategies utilized over time by RPs to construct their own professional identities. The definitions of the key strategies utilized to cognitively construct and align self-attributes with professional aspirations in each phase are provided in Table 2.

**Table 2:** Definitions of key strategies utilized in each phase.

IBCS phases	Key strategy	Definition
1	Isolating contrasting self-attributes	Cognitively *separating* contrasting self-attributes within self to align with the perceived attributes of non-AEC professional aspirations.
2	Aggregating contrasting self-attributes	Cognitively *bundling* contrasting self-attributes within self to align with the perceived attributes of broad AEC professional aspirations
3	Ranking contrasting self-attributes	Cognitively *ordering* contrasting self-attributes within self to align with the perceived attributes of specific AEC professional aspirations.
4	Blending contrasting self-attributes	Cognitively *integrating* contrasting self-attributes within self to align with the perceived attributes of AEC professional specialization aspirations.

In Phase 4, a more detailed visualization is provided in Figure 1 to highlight cognitive and behavioral processes that contribute to AEC-PID in undergraduate women, while navigating gendered tensions in undergraduate AEC contexts. Evolving engagements drive the iterative cognitive specification and re-specification of the contrasting self-attributes that must be blended to become an AEC woman professional. Together, these cognitive specifications form the *Blending Contrasting Self-attributes (BCS) standard* (Stets and Burke, 2025) shown in Figure 1 as the *woman with the hard hat and BCS shirt*. The BCS standard captures RPs subjective and unique conceptualizations of the requirements and expectations associated with being a woman professional. Therefore, RPs iteratively assess similarities (*right tornado*) between self-attributes and BCS standard during identity affirming situations and assess differences (*left tornado*) during identity conflicting situations. Considering that there are variations in RPs’ self-attributes, agency, and meaningful engagements within undergraduate AEC contexts, RPs express varied types and intensities of discrepancies. Considering that discrepancy types and intensities vary across RPs, some RPs elevate personal self-attributes over the BCS standard while others elevate BCS standard over personal identity. These varied and agential internal negotiations influence variations in subsequent enactments, re-specification of self-attributes, and integration of personal and professional identities to commit to AEC professions. We utilize three different trajectories (*terminal rectangles colored light green, deep green, and red*) to characterize, explain, and cluster the varied negotiation mechanisms and enactments utilized for resolving discrepancies and committing to AEC professional specialization aspirations. Many RPs blend contrasting self-attributes, resolve tolerable discrepancies, and optimally integrate personal and professional identities to conform and remain within the boundaries of AEC professions. However, with significant and intolerable discrepancies, other RPs demonstrate agency by altering or breaking out of AEC professional boundaries to accommodate sub-optimal or disrupt non-optimal personal-professional integration. We utilize subsequent paragraphs to elaborate our results and substantiate with direct quotations. Results for research questions 1 are captured in phases 1 to 3 while results for research question 2 are captured in phase 4. For confidentiality, RPs are assigned pseudonyms and described only by the three broad AEC disciplines.

### 3.1 Phase 1: isolating contrasting self-attributes to align with the attributes of non-AEC professions during mainstream engagements leads to the pursuit of more advanced STEM and arts engagements

Our analysis revealed that due to lack of awareness and misconceptions about women in AEC professions, RPs had non-AEC professional aspirations prior to developing AEC aspirations. Evolving non-profession related mainstream engagements (causal conditions) in early family, educational, and media contexts (contexts) contributed to their specification of master personal identities as fundamental to the origination of their professional identities. Feedback from socializers and personal enjoyment during formal (*N* = 35), informal (*N* = 44) and gendered (*N* = 29) mainstream engagements made RPs aware of their multiple and contrasting inherent self-attributes. Through meanings from these mainstream engagements (e.g., play, sports, school, media), RPs began to describe themselves as possessing contrasting self-attributes to include problem-solving (creative/analytical/practical), analytical (argumentative/non-argumentative), social (altruistic/competitive), interdependent (dependent/independent), object interests (natural/man-made), space interests (known/unknown), and gender (girly, tom-boy, in-between) attributes. These self-attributes are characterized as inherent because RPs described them as their natural inclinations. While variations existed across RPs, these self-attributes were broadly clustered into the three dimensions of interests, abilities, and resilience. RPs also gained awareness and understanding of opportunities, threats, stereotypes, and challenges within these contexts. Through school, play, and family engagements, engineering RP, Hagar, became aware of her inherent contrasting problem-solving (creative/analytical) and social (competitive, altruistic) and specified her self-attributes as described:

I like to dance…I love [*interest*] math and science…I was good [*ability*] in math…Art… I love it…I was recognized by my teacher for my drawing…but, I still really liked math…I was the only girl on the team… at first, I’m like yea, because it’s like I’m standing up…, but eventually I’m just like ‘Ok, we need some more girls’… watching the guys play first… then I would go ahead and sub in [*resilience*]…they [siblings] are really into music and art and we would stay home and make beats…I would mainly say T.V. but growing up with a lot of siblings you also see like many of them get sick… Get a cold or something like that so we would be like ‘I’ll help you out.’… ‘Ok, I’ll grab the tea,’ you know, ‘make you feel better.’

Notably, through sports interactions with boys, RPs like Hagar developed the resilience to bounce back from gendered tensions in MM contexts. These important contrasting self-attributes formed the master personal identities of RPs and agentially directed subsequent decisions and engagements. Over time, with guidance from socializers, RPs transitioned to varied and contrasting profession-relevant mainstream engagements (causal conditions) in introductory non-AEC STEM, Arts, and Humanities contexts (contexts). These engagements increased their awareness and understanding of the attributes of several non-AEC professions. Considering that they were engaged in multiple contrasting engagements (e.g., extracurricular, tv shows), RPs often conducted iterative person-profession fit assessments (intervening conditions) by evaluating the similarities and differences between their self-attributes and the attributes of non-AEC professions. When they perceived fit, they aligned their self-attributes with these professional attributes, and these shaped the development of their earliest professional aspirations (Phenomenon). RPs re-specified (Strategy) their inherent self-attributes to include profession-related attributes. Through media engagements, Hagar, re-specified her argumentative analytical interests by adding altruism and aligning to her lawyer aspirations as stated:

Lawyer…I really like the aspect of arguing…I thought in elementary school…people need my help so that I can get them out of trouble… definitely say on T.V.…like Law and Order…Middle school, I really, really wanted to be a dancer…I love dancing.

Like Hagar, considering RPs possessed multiple contrasting self-attributes and experienced diverse contrasting engagements, they tended to isolate and align re-specified self-attributes with a single aspiration at any given time. We describe this as the *isolating contrasting self attributes* strategy (strategy) as defined in Table 2. Although Hagar possessed both creative and analytical self-attributes, she isolated her argumentative analytical self-attribute to align with her lawyer aspirations and isolated her creative self-attribute to align with her dance aspirations. Over time, RPs shifted among several non-AEC professional aspirations. Like Hagar, RPs (*N* = 67) applied this strategy to interests which we coded as idealistic because of limited exploration and heavy reliance on emotions and intuition. Like RPs (*N* = 56) who applied this strategy to abilities, Tabitha, a construction RP, aligned contrasting creative, analytical and practical abilities with non-AEC STEM, Arts, and Humanities aspirations as explained:

… in elementary school, I wanted to be a photographer. Then I wanted to be a historian. Then I wanted to be an artist. Then I wanted to be a scientist. Then I wanted to be a dancer. … Gymnastics…I wanted to be a whole bunch of things…We had block parties he [Dad] would be the photographer taking pictures… I was in art club, so I thought that’s what I wanted to do. I could draw really well. We had science classes…I thought it was interesting so I thought oh I could be a scientist. I could be a neurosurgeon. It was very hands on… I was enrolled in a dance school so I was like I can do dance until I did not like it anymore… She [Mom] put us in a lot of different activities. She likes to call it mainstreaming us… I did really well in art and English and history and stuff… my dad, he’s an African American history teacher. Basically, I just stuck with the art clubs and the science clubs up until high school… I went to a magnet high school. It was called *Engineer and Science*.

We coded these as generic abilities because of associations with a wide range of STEM, Arts, and humanities disciplines. We note here that RPs descriptions suggested limited agency during this phase because like Tabitha’s mom, other socializers were heavily involved in directing RPs’ subsequent engagements. Tabitha noted that her mom utilized “mainstreaming” to express the behavior of socializers who connect RPs to engagements to help RPs conform to societal standards.

To sustain STEM aspirations, RPs developed diversified resilience (*N* = 44) by sourcing varied strategies to recover from stereotyped threats and underrepresentation that produce gendered tensions in pre-college engineering courses, family and other social engagements. Resilience was contrasting because RPs utilized both feminine and masculine sources, as well as dependent and independent strategies to resist threats. Rachel, an engineering RP, applied dependent resilience by relying on her parents to resist stereotyped comments from her grandparents. Also, she applied independent resilience by expressing personal agency with *doctor* (masculine) or *sit at home* (feminine) choices as explained:

They [Grandparents] wanted them [women] to stay at home and like have children…I would be sitting at the table and they’d be like, ‘what do you want to be when you grow up?’ and I’d be like, ‘a doctor, and I’d like to go to Oxford as well.’ And they’d be like, ‘well, what about a mommy?’… and my parents would be like, it’s perfectly fine for her to want to be a doctor.’ And they are like, I’m just saying she should want to be a mother.’ So, I do remember that. Oh, feeling like…I could do whatever I want. I could do both. I could do neither and just like sit at home.

Over time, to advance towards aspirations, like Tabitha, RPs pursued more advanced STEM and ARTS engagements (outcome) in substantive STEM and ARTS contexts, and these became the causal conditions and contexts for the next phase of the IBCS theory—phase 2.

### 3.2 Phase 2: aggregating contrasting self-attributes to align with the attributes of broad AEC professions during AEC-infused STEM and ARTs engagement leads to pursuit of AEC exploration engagements

Through participation in advanced STEM and ARTS engagements in substantive STEM and ARTS contexts (contexts), RPs were drawn into AEC-infused STEM and ARTS engagements (causal conditions) within this context and developed broad AEC aspirations (Phenomenon). These engagements were characterized as AEC-infused because they were STEM or ARTS engagements that introduced RPs to basic physical or virtual components of built environments to include AEC objects, processes, and socializers. We coded AEC-infused engagements as remote (*N* = 56), proximal (*N* = 35), and immersive (*N* = 11). Through positive feedback from authority figures (e.g., teachers, family members) during AEC-infused visual-kinesthetic engagements (e.g., observations, formal STEM and ARTS education, informal pre-college programs, do-it-yourself projects, games/toys, media, and industrial experiences), RPs began to elevate man-made objects (e.g., buildings, roads) over natural objects (e.g., humans). Also, they began to elevate known spaces (e.g., outside spaces on earth) over unknown spaces (e.g., outer space) because known spaces were more practical. RPs increased in their awareness, understanding, and passion for the attributes of AEC professions as they could utilize their contrasting self-attributes, particularly creative, analytical, and practical problem-solving self-attributes. With increased understanding of the contrasting attributes of AEC professions, RPs conducted iterative person-profession fit assessments (intervening conditions) and evaluated differences and similarities between their contrasting self-attributes and the attributes of broad AEC professions. Perceiving fit, RPs re-specified (strategy) self-attributes by adding AEC attributes. Through a high-school course, Ariel, an engineering RP, added terms like *esthetic* and *design* to her contrasting creative and mathematical self-attributes and aligned with architecture and engineering aspirations as described:

I wanted to be an engineer…I took an architecture class because they kind of combined architecture and engineering in my high school…We were in AutoCAD…We were building different things, and I really enjoyed … like the design and so I knew that I want to do something creative, but also practical…I really enjoyed multiple things about architecture… I really love the videos we would watch in my engineering high school class… creative can also apply to like math … just interesting ways of solving things…it’s the esthetic part to it, for me, usually like arranging things in a certain way.

Like Ariel, RPs began to cognitively bundle several contrasting self-attributes within self to initiate alignment with the perceived contrasting attributes of broad AEC professional aspirations. We coded this as the *aggregating contrasting self-attributes* strategy (strategy) as defined in Table 2. Like RPs (*N* = 68) who applied this strategy to interests, Ariel aggregated practical, creative, and analytical interests and initiated alignment with broad AEC aspirations. These interests are characterized as *semi-idealistic* because they mostly emerged through interactions with modeled, simulated, and remote AEC processes in substantive STEM and ARTS contexts. Aspirations are characterized as broad because RPs had two or more AEC aspirations, often architecture and engineering, as high school tended to combine architecture and engineering engagements. We note there that construction professional aspirations were rare in phase 2 because of low visibility in pre-college contexts. Like RPs (*N* = 67) who applied this aggregating contrasting self-attributes strategy to abilities, an architecture and engineering RP, Lois, noted that her contrasting art and math abilities made her different from her two brothers who were on opposite sides of the problem-solving spectrum as stated:

I did pretty well on that [Math] … My dad was also [like me] always a really good drawer … which is why I ended up doing Architecture and Architectural Engineering because I like both the art and the math and science part… my two younger brothers, they are kind of on opposite spectrums. One is more like his brain thinks more artsy out of the box, while the other one is very like straightforward math science…they have a hard time like communicating about homework and asking questions between the two of them…I can help them because I understand how their brain thinks…. We drafted by hand, but then we use SolidWorks for the 3D Modeling… I had the most experience because of using tools at home and using them in middle school.

Lois also had practical skills and could use both physical and digital tools. The aggregation of these self-attributes increased her self-efficacy and initiated her alignment with broad AEC aspirations. These abilities are characterized as *pre-requisite* because they are a prior condition for AEC aspirations that demand basic creative, analytical, and practical problem-solving abilities.

To sustain AEC aspirations and reduce gendered tensions in AEC educational and industrial environments, RPs re-specified diversified resilience (*N* = 40) to include AEC-related contexts. Sheerah, a construction RP, demonstrated *dependent resilience* by relying on her grandfather’s defense to recover from site workers’ stereotyped comments as explained:

When I first started working there, his [Grandfather] other workers would try to like to help me… and my grandfather would be like ‘No, let her do it!…she can definitely do it!’ so that gave me confidence … I like to get my nails done… everybody would always be like…‘you are going to mess them up and get them all dirty.’ But I would not care… I do not mind getting dirty at all….

She exhibited contrasting *independent resilience* by recovering from stereotyped comments and agentially making her own *nails* (feminine) and *dirty* (masculine) choices.

Over time, to gain more understanding of AEC professions, RPs began to pursue AEC exploration engagements (outcome) in substantive AEC contexts as described by Shalom, an engineering RP who explored different types of engineering:

I was speaking with my AP calculus teacher, and she had a degree in, well she was studying to be in architecture … so she was kind of like, … ‘you can be an engineer’ … I started doing my own research on engineering…mechanical, electrical, civil, and then I saw environmental.

Over time, to gain a more advanced understanding of AEC professions, RPs agentially pursued AEC exploration engagements (outcome) in substantive AEC contexts and these became the causal conditions and contexts for the next phase of the IBCS theory—phase 3.

### 3.3 Phase 3: ranking contrasting self-attributes to align with the attributes of specific AEC professions during AEC exploration engagements leads to AEC undergraduate enrollment

With growing interests in broad AEC professions, RPs wanted to know more about AEC professions. Therefore, they participated in AEC exploration engagements (causal conditions) in substantive AEC contexts (contexts). Explorations were conducted dependently (*N* = 42) and independently (X = 36). We coded two levels of explorations. The first level involved comparison of non-AEC and AEC professions, and the second level involved comparison among AEC professions, programs, and institutions. Meanings from these explorations increased RPs’ understandings of the attributes of AEC professions. During iterative person-profession fit assessments (intervening conditions), self, social, and structural comparisons occurred and revealed similarities and differences among AEC, non-AEC, and self attributes. Becoming more informed, RPs perceived misfit with non-AEC professions and fully abandoned earlier non-AEC aspirations and strengthened their alignment with broad AEC professions (Phenomenon). RPs re-specified (strategies) self-attributes to include outcome expectations and professional attributes. With first level exploration, some RPs perceived outcome expectation discrepancies like impracticality, financial hardships, and long educational periods and abandoned non-AEC aspirations like astronaut, artist, and doctor, respectively. RPs like Ruby, an architecture RP, re-specified her creative interests by appending financial interests and switched from artist to engineering aspirations. Interests are characterized as *semi-realistic* because RPs were driven by facts and real-life experiences in substantive AEC contexts. RPs utilized terms like *more attainable* that captured increments in reality as Ariel, an engineering RP, who switched from astronaut to AEC aspirations expressed:

I wanted to be like an astronaut… it seemed unattainable… that’s why I lean more towards architectural side because it’s something that, you know, I see every day and it seems more attainable and like more real.

RPs began to cognitively arrange contrasting self-attributes in a hierarchical order and strengthened the alignment of their highest ranked self-attributes with broad AEC aspirations. We describe this as the *ranking contrasting self-attributes* strategy (strategy) as defined in Table 2. Some RPs ranked creative above practical interests and fully switched from robotics and mechanical engineering to AEC aspirations. Others ranked analytical above creative self-attributes, and switched from fine, performing, and culinary arts to AEC aspirations. Applying the ranking strategy (strategy) during second level explorations which compared AEC professions, RPs aligned the highest ranked contrasting self-attributes with corresponding specific AEC professions. Like RPs (*N* = 12) who assigned the highest rank to creative interests, an architecture RP, Ruby, chose architecture over engineering as explained:

I decided engineer was definitely not for me because I excelled in math, but I knew math wasn’t something I really wanted to do … I loved drafting and coming up with problem solving ideas and being creative… the perfect mixture is architecture.

Like RPs (*N* = 21) who assigned the highest rank to analytical interests, Jennifer chose engineering over architecture. She also wanted to avoid the sleepless lifestyles associated with architecture professions. Few RPs (*N* = 6), like Lois, had two equally ranked contrasting creative and analytical problem-solving interests and therefore enrolled in a dual architecture and engineering program. RPs applied this ranking strategy to abilities. Abilities were re-specified to include formal undergraduate qualification requirements such as SAT standardized tests for college readiness. Therefore, abilities are characterized as *qualifying* because they are needed to enroll in undergraduate programs. Missing qualifying requirements for engineering, few RPs switched to construction programs which had lower qualifying requirements.

To sustain AEC aspirations and recover from gendered tensions, RPs added *modeled resilience* to diversified resilience. They gained resilience by observing or interacting with female role-models as explained by Namangolowa, a construction RP:

She was the first person I had met that had been in building construction and who was a woman, who was of color, and she was successful. So, I was like ‘okay, this is something that I could actually do.’

Over time, to gain qualifications to become professionals in specific AEC professions, RPs agentially pursued AEC expert engagements (outcome) in undergraduate AEC program contexts and these became the causal conditions and contexts for the next phase of the IBCS theory—phase 4.

### 3.4 Phase 4: blending contrasting self-attributes to align with the attributes of AEC profession specializations during AEC expert engagements leads to commitment to become an AEC woman professional

During phase 4, RPs participated in AEC-expert engagements (causal conditions) in AEC undergraduate education (context). The complexity of engagements transitioned from basic gateway (e.g., orientation, general education courses, AEC introductory courses) to complex academic (e.g., advanced AEC courses, labs, studios, research). Also, engagements became increasingly realistic as RPs transitioned from academic to more experiential (e.g., internships) engagements in AEC industry contexts. Engagements increased understanding of the attributes of AEC professions. RPs described navigating increasingly gendered AEC engagements and tensions (e.g., underrepresentation, marginalization) especially as they transitioned from academic to industrial engagements. They described gendered tensions and expressed deep concerns about the downgrading of women abilities, working harder than their male counterparts, and potential barriers to their career progression as narrated by Martha, an engineering RP:

#1 I’m a female in this industry and #2. I’m African American. So, I feel like those two are going to be like the biggest problems… because of the way the industry is set up now. It is more of a male, Caucasian industry. So, because I’m other than just my skin color, because I’m a female. You know, females are usually downgraded in certain things. So, me trying to get in this industry I feel is going to be a little tougher than my male counterparts, trying to get into this industry as well… So yes, my concern beyond that is getting up in the industry. So, now that I made it, can I rise up to those CEO roles that the male-Caucasian people have conquered for years? And will they accept me when I do get up there? Because that is the plan to get up there. So, when I do get up there, will they accept me?

Due to these gendered and racial challenges, Martha felt that she had to be tougher to persist to become an AEC woman professional. Meanings from new engagements informed the cognitive specification of the contrasting attributes required to become an ideal AEC woman professional. Together, these specifications formed the cognitive AEC woman professional identity standard. We caption this as the *Blending Contrasting Self-attributes* (BCS) standard as it drove RPs to consciously or unconsciously cognitively blend contrasting self-attributes (strategy) to align with the attributes of AEC professions. Over time, varied meanings led to the re-specification (strategy) of different types and intensities of attributes within each RP’s standard. Yet, regardless of RP or discipline, each standard consisted of different types of contrasting self-attributes which we clustered and coded as *realistic interests*, *professional abilities*, and *transformational resilience* (Table 3).

**Table 3:** Focused and open codes for the *Blending Contrasting Self-Attributes (BCS)* category.

Category	Focused codes	Open codes (properties and dimensions)
Blending contrasting self-attributes	Blending contrasting realistic interests	Blending Origination and Replication creative problem-solving interests. Blending Numerical and Non-numerical analytical problem-solving interests. Blending Physical and Digital practical problem-solving interests. Blending Unstructured and Structured problem-solving interests. Blending Theoretical and Practical problem-solving interests. Blending Hard and Soft skills interests. Blending Independent and Interdependent interactional interests. Blending Outdoor and Indoor AEC space interests. Blending In person and Virtual space interests. Blending Large and Small AEC object interests. Blending Physical and Digital AEC object interests. Blending Intra-disciplinary and Inter-disciplinary interactional interests. Blending AEC and non-AEC interests.
	Blending contrasting professional abilities	Blending Origination and Replication creative problem-solving abilities. Blending Numerical and Non-numerical analytical problem-solving abilities. Blending Physical and Digital practical problem-solving abilities. Blending Hard and Soft skills.
	Blending contrasting transformational resilience	Blending Feminine and Masculine resilience self-attributes. Blending Female and Male networking resilience self-attributes.

Like previous phases, RPs iteratively conducted person-BC standard fit assessments (intervening conditions) and re-specified self-attributes by utilizing realistic AEC expert terms to describe themselves. Nevertheless, most RPs encountered person-standard fit discrepancies that activated internal negotiations. Initially, RPs endeavored to resolve discrepancies by elevating the BCS standard over their personal identities and engaged in *beneficiary* (receiving support – e.g., tutee, member, mentee) roles. Over time, some RPs progressed from beneficiary roles into *benefactress* (giving support – e.g., tutor, leader, mentor) roles. Applying the BCS strategy to her contrasting and realistic outdoor/indoor interests, Martha, an engineering RP, explained how meanings from job interview engagements caused her to increase her ranking of outdoor space interests and re-specify her BCS standard as stated:

I have my times, where I’m like, ‘oh yeah, I could climb up the ladder. I can play as dirt.’ and then, I have other times, ‘I’m like, I don’t want to do that. I don’t want to be out in the field’ … but … they [interviewers] are always telling me, ‘you’re going to be in the field’, when, I’m, you know, doing these interviews. The first thing, I’m saying is ‘okay, I have to get over that girly girly mind set of *I don’t want to do that. I don’t want to be in the field*, if I want to do this, what I want my profession to be,’ so I had to push myself to kind of step out of the girly girl mindset, sometimes, because this is my career… I am definitely getting the experience of office, I’m getting field experience, so uhm I’m definitely learning from both sides… So, I was able to see basically what I will be doing when I do get into my profession… for the jobs that I was on, I was able to be in the office, but I was able to go to the field whenever I wanted to … I was able still see the process and not just be stuck behind the computer…I would honestly say that 75% will be on site and 25 will be in the office.

Notably, Martha associated her natural indoor space interests with being feminine and utilized the term “girly” to describe this mindset. This captures the gendered meanings associated with some AEC engagements and contexts. Her person-standard fit assessment revealed a discrepancy because her inconsistent outdoor interests were less than required by her BCS standard and this activated internal negotiations.

During negotiations, Martha elevated the BCS standard above her personal identity and resolved this interest discrepancy by eliminating her feminine mindset to meet the outdoor space interest requirements of her BCS standard. She enacted this decision during her internship (*beneficiary role*) and learned from contrasting office and field (contrasting contexts) work. Martha re-specified her interests by assigning a higher percentage to her outdoor (75%) over indoor (25%) interests. Similarly, Martha also described the blending of contrasting origination (*birthing novel ideas*) and replication (*copying existing ideas*) creative self-attributes in a co-ed student organization concrete canoe competition project. These interests are characterized as *realistic* because RPs have direct and real-life interactions with AEC objects, processes, and experts. The successful resolution of interest discrepancies drove motivation to persist.

In applying the BCS strategy to contrasting professional abilities, Martha re-specified her soft skills by transitioning from hesitancy to presenting to AEC professionals. She re-specified hard skills by serving as a leader (*benefactress role*) in materials labs. During the co-ed canoe project, she also blended numerical and non-numerical analytical abilities. These abilities are characterized as *professional* because competencies are developed for professional practice and licensure. Abilities sometimes raised gendered tensions because women’s knowledge and abilities tended to be downgraded. Therefore, RPs were driven to work harder and this in turn increased their self-efficacy and motivation to perform as professionals as noted by Martha:

…I feel like I’m one of the few that’s going to show the rest of these males that a female can do it just like they can. So at that point, my goal is to outdo what they do, to let them know, like, ‘hey, this isn’t just a male dominated career. Like, females can do it, too.’ So I just feel like that’s more of a drive and push for me to do beyond what I know I can do. Just to prove them wrong. Yeah… And I feel that from this experience, just being around like my, my male friends. We will, will go places, or will do something and they do not expect me to have the knowledge or the ability to do it just because I’m a female. And I had to tell them all the time, ‘just because I’m a girl, that doesn’t mean that I can’t do the same thing that you do… the way I grew up, I was always helping put, to mount TV’s, I was helping to mount TV. I was helping to move furniture and things like that. And they tend to think, because I’m a female, ‘you can’t pick this up, it’s too heavy or you can’t do this because you don’t know’. And I’m like, well, the way I grew up, they, ‘we’re all the same’. So, I’m always lifting heavy things as well. It’s not just a male type thing.

Here, Martha signals the importance of engaging girls in a variety of activities to develop a range of self-attributes to support their aspirations. Martha resolved heightened gendered tensions and resilience discrepancies, by adding *transformational resilience* to diversified resilience. RPs expressed both differences and similarities with male peers and implemented various contrasting strategies to bounce back from different gendered situations. RPs often assessed situations and results from their assessments informed agentic decisions to conform or resist stereotyped expectations and structural barriers. Like Martha who blended contrasting female and male networking by joining both co-ed and female-only student organizations, RPs blended contrasting attributes to function and persist. Martha noted that co-ed organizations offered more professional development opportunities like concrete canoe competitions while female-only organizations offered more community support and opportunities for altruism as explained:

…being involved with not only the women on campus, but also the men … we are all going through the same struggles…I was able to talk to men and women and get their perspective on the profession … We did the concrete canoe…[Co-ed organization] gives more opportunity than [Female only organization]. [Female only organization] is more of a community, like, I will say that the women group in lifetime is, I believe it’s more of a community …if I remember correctly, I do not think there’s any competition or any conference that they go to… It’s more of just oh, we are gonna have a general body meeting…and we are gonna do community service. But, … I guess what I was looking for was the engineering part of the organization.

A full listing of the focused codes that contributed to the blending contrasting self-attributes category is shown in Table 3. Like Martha, the successful resolution of discrepancies increased AEC professional abilities, interests, and resilience and drove self-efficacy, confidence, motivation, and sense of belonging to AEC professions. Considering that resilience was often related to gendered tensions from downgraded abilities and interests, the resolutions of abilities and interest discrepancies was important to RPs. Consequently, resilience developed over time and RPs increasingly positioned themselves as equal to their male peers and transitioned from beneficiary to benefactress roles—a significant indicator of AEC-PID as RPs expressed thinking, feeling, and acting as professionals (Phenomenon). Developing resilience from gendered tensions inspired RPs to make strong commitments to transform AEC professions. Few RPs adopted a more individualistic approach to transform AEC professions by representation which meant being present and self-displaying as competent and interested AEC woman professionals. Other RPs adopted both individualistic and collectivist approaches. The collectivist approach involved bringing other girls and women into AEC contexts to develop competencies to influence AEC contexts. These RPs believed that women could contribute their unique problem-solving and altruistic talents to diversify innovation and inclusion in AEC engagements and contexts as explained by Martha:

I did used to have a concern about choosing this profession and going into it as a black female … there is only 1.6% of us … I hope to recruit more into the 1.6% … male AEC students … have more of … a foot in the door into this type of industry than females because we do turn to get seen as that girly girly type of person, when well it’s not completely true half of the time … As women in the AEC program, we are just positioning ourselves to make changes in the companies that we plan on working for in the future and not even just in the company, but in our communities and in the country …my influences come from modeling myself showing to younger females and people… looking at the AEC profession, you know, ‘if she can do, I can do it’… No matter your race, no matter your religion, everyone’s in the field … I noticed that it was male dominated, um so I wanted to kind of even it out a little bit or take over the E-board but as females… I love helping people… I can explain it to them and they leave with a smile on their face… we are able to get a spot on the board, so that, we can basically be an influence and mentor to the new members coming in.

With no unified concept to capture this unique resilience mechanism, we coined the new *womfessionalization concept.* This concept combines the first three letters of the word “*woman*” and the last 16 letters of the word “*professionalization*.” Our new “*womfessionalization*” concept captures a unique and collectivist resilience mechanism which STEM women with professional self-efficacy and motivation utilize to resolve strong gendered tensions by pursuing influential problem-solving and altruistic roles to advance innovation and inclusion for all within MM STEM contexts.

The progressive integration of personal and BCS standard attributes strengthened RPs’ AEC-PID and sense of becoming AEC woman professionals (Phenomenon). Considering individual differences exist among AEC women undergraduates who also encountered different engagements in different settings, they experienced different types and intensities of personal-standard fit discrepancies, and a variety of mechanisms were utilized to resolve and integrate personal and professional identities. We found three types (*interest, ability, resilience*) and intensities (*tolerable, significant, intolerable*) of BCS discrepancies that generated tensions in AEC-PID. Furthermore, we utilize three BCS trajectories (*Conformers, Alterers, Breakers*) to explain the varied negotiation and enactment mechanisms utilized by RPs to resolve these discrepancies.

1. *Conformers (N = 47)* encountered tolerable BCS discrepancies and made full and long-term commitments to professions within the boundaries of AEC domains. This is because they achieved the strongest contrasting self-attribute blends and elevated the standard over personal identities. In resolving tolerable discrepancies, they limited the reconsideration of AEC aspirations and immersed themselves in AEC offerings. They enacted AEC strategies that led to the re-specification of self-attributes to meet BC standards and transitioned into benefactress roles. We identified three mechanisms that Conformers utilized for resolving discrepancies: (a) The *Refinement* mechanism (*N* = 35) involved slight adjustments to self-attributes and the pursuit of specializations within original AEC program boundaries. With origination over replication, independent over interdependent, and female over male networking during a project management internship, Rachel, an engineering RP, shifted to structural engineering as explained:

project management. I shadowed um an engineer on a construction site, my freshman year. I thought it was super interesting … amazing like solving a puzzle a little bit you know, creating schedules, budgeting I really, really liked it… you get to move around a little bit more, get to get out of the office. You get to interact with a lot of different people so that part really appealed to me… structural engineering … because I’ll be in fewer areas where I would be in like a space dominated by men …. as a project management intern and working construction … 90% of my job was just calling people … I found it exhausting…whenever I would get a chance to work with, you know, structural engineers … I was a little bit more excited … It’s just nice to be there at the beginning of something … it felt more like you were trying to put a puzzle together… Construction was like you are handed the already finished bubble.

With outdoor over indoor and interdependent over independent interests during an internship, Yosheeka, a construction RP shifted from project management to superintendent aspirations. With interest in small details over large scale, Esther, an Architecture RP chose residential over commercial specialization. (b) The *Reformulation* mechanism (*N* = 12) involved the revision of self-attributes and shifts from original to non-original aspirations within AEC domains. Engineering RPs with numerical analytical ability discrepancies (*N* = 3) were advised to switch to construction as explained by Abigail who maintained original engineering interests despite math ability challenges. Others encountered interest discrepancies and shifted (*N* = 3) or intended to shift (*N* = 6) to non-original AEC aspirations. With creative over analytical interests, an engineering RP, Hagar, intends to shift from civil to architectural engineering through graduate education. (c) The *Womfessionalization* mechanism was exhibited by Tabitha, a construction RP, who developed resilience by focusing on the unique problem solving and altruistic attributes of women and expressed a strong desire to place the woman’s touch and transform AEC spaces through more diversity innovation and accessibility for all as explained:

I think girls bring like their eye detail like they know when stuff looks good, well guys I think they just throw it together like you need like a woman’s touch on buildings so it could look good… We’re putting ourselves in these positions to transform the spaces … women are very good authority figures because they had this sense of compassion and those soft skills… Just women like, how to make the building more women friendly or how to make the building like accessible for everybody, like they get things that will be needed in the building like. When we might need this for … we are building that… nobody else does it. So just bringing another perspective. How things should be done… should look like.

Like Tabitha, Angelina, an engineering RP, emphasized that women are out-of-the-box thinkers with new problem-solving ideas to contribute to innovation. RPs desire to transform AEC educational and professional spaces, as well as their family and social spaces. Conformers graduated or intended to graduate from AEC programs as they achieved optimal person-standard integration with commitment to become women professionals with full AEC membership.

1. *Alterers (N = 15)* encountered significant discrepancies and made partial and short-term commitments to professions within the boundaries of the AEC domain. This is because they achieved medium strength contrasting self-attribute blends and tended to elevate their personal identities over the standard. They resolved discrepancies by reconsidering the standard, immersing in AEC and non-AEC offerings, and enacting AEC and non-AEC strategies. We identified three negotiation mechanisms: (a) The *Periphralization* mechanism (*N* = 10) involved distancing themselves from the standard by maintaining intentions to pursue non-AEC professions that are closely related to the AEC industry. With non-numerical over numerical and interdependent over independent interests, an engineering RP, Debra considered legal consulting aspirations as explained:

That’s like also something that I miss in engineering… I do not write essays anymore, and I do not be challenged, like [that] side of my brain anymore and so I think that this, like, legal consulting… the application of legal components to engineering field.

(b) The *Temporalization* mechanism (*N* = 5) involved distancing themselves from the standard by maintaining intentions to limit time commitments to AEC professions. With feminine over masculine preferences, Jennifer, an engineering RP, enrolled in language and business minors and planned to switch to business aspirations as noted.

I’m thinking of going to more business side… I have the French and Business minors … I’m more on the girly girl side… when I was interning, I was always the most dressed up … other girls that work there were wearing like chicos and super dressed down… I’ve always liked the style fashion aspect and that’s … very necessary in the business world…I guess I would be considered a role model for other girls unintentionally. I feel like the people that are interested will be interested and the people that aren’t interested, aren’t interested in AEC… So I guess if I was to do that and kind of talk about what I did and maybe the girls in the class would be like, oh, I can do that to. But I do not necessarily think of it like, oh, I’m a girl in AEC. I’m more like, oh, I’m a civil engineer.

Alterers perceived themselves as different from other AEC students to include AEC women as noted by Jennifer. Despite strong abilities and interests, Ariel, an engineering RP, preferred female over male networking and person over engineer identity causing resilience discrepancies. Attributed to her plus-size and negative boys’ club experiences during an internship, Ariel switched from HVAC specialization to non-AEC aspirations. (c) *Womfessionalization* was utilized by Alterers like Ariel. With concerns arising during late undergraduate years, Alterers graduated or intended to graduate from original AEC programs while exploring AEC options and non-AEC alternatives. They accommodated sub-optimal person-standard integration with AEC membership restrictions.

1. *Breakers (N = 3)* encountered intolerable discrepancies, withdrew from AEC aspirations, and made no commitments to professions within the boundaries of the AEC domain. They made full and long-term commitments to non-AEC professions which were beyond the boundaries of AEC domains. This is because they achieved negligible contrasting self-attribute blends and tended to elevate personal identities over the standard. They had tried to resolve discrepancies by reconsidering the standard, immersing in AEC and non-AEC offerings, and enacting AEC and non-AEC strategies. With limited success during early undergraduate years, they remained in beneficiary roles and switched early into non-AEC programs. Applying the *Withdrawal* mechanism, Breakers rejected the standard, withdrew from AEC aspirations, and switched to non-AEC programs. With replication over origination creative problem-solving interests, and small over large object interests, Alyssa, an architecture and engineering RP switched to chemical engineering as noted:

I’m definitely more creative in the observational manner. I like to draw while looking at something …. I do not like just getting a blank canvas again and being told like a few rules and guideline…. Architecture was too demanding for me in the creative and hands on aspect…’ after taking a chemistry class, …I changed my major from Architectural Engineering to Chemical Engineering…I enjoy having some kind of format and structure … I originally thought I’d like to work with large things, the large buildings… after being in AEC, I realized that those large things have a lot of detail in them and going back to like HVAC, those details do not really interest me… I prefer to work on smaller things.

On the contrary, with origination over replication, and digital over physical interests, Leah, an architecture RP, switched to screenwriting animation as she described:

I’m a senior screenwriting animation major … It’s just more interesting to me … because the architecture, like, I only did one semester. I actually had like a 3.5 GPA … I decided not to pursue it … I thought there would be more art involved… I was gluing together blocks and making physical projects and stuff but it’s like in Animation … you could be more creative and make what you see in your head …. I like digital… more creative freedom… I’m not, like in love with buildings.

They attributed switches to unmet expectations which were driven by earlier unrealistic assumptions about AEC professions as noted by Leah. Breakers disrupted person-standard integration and retained non-membership status.

## 4 Discussion

We extend PID literature by introducing our novel multi-dimensional, multi-phase, multi-context, and multi-disciplinary, *From Isolating to Blending Contrasting Self-Attributes (IBCS)* grounded theory. Adopting constructivist perspectives and a longitudinal approach, we provide a more nuanced and holistic understanding of agency and trajectories in AEC-PID processes in AEC women undergraduates. Our IBCS model captures the progressive range of important self-attributes, meaningful engagements, agentic strategies, and relevant contexts that interact and evolve as AEC women undergraduates agentially construct their own sense of becoming AEC women professionals, while navigating gendered tensions. In subsequent paragraphs, we explain our theoretical, methodological, and practical contributions.

### 4.1 Theoretical contributions

First, the IBCS theory aligns and links well-established identity and role congruity frameworks. It aligns with social identity frameworks (Tajfel and Turner, 2004; Crocetti et al., 2023), by explaining how increasingly meaningful engagements within AEC contexts provide the activities, resources, and conditions to progressively develop self-attributes and facilitate AEC-PID. Aligning with personal identity frameworks, this theory explains how multiple self-attributes originate, iteratively evolve, get structured, and integrate to become the components or content of AEC professional identities to facilitate AEC-PID (Stets and Burke, 2025; Crocetti et al., 2023). By highlighting how systemic gendered inequalities and structured barriers drive conflicts among woman, student, and professional identities to cause gendered tensions in PID, the IBCS theory aligns with role congruity theories (Eagly and Karau, 2002). Lastly, this IBCS model aligns with constructivist perspectives and agency frameworks because we explain the unique and varied identity negotiation strategies and mechanisms actively utilized by women to resolve personal-professional identity fit discrepancies to commit to becoming women professionals (Chávez et al., 2023; McCall et al., 2021; Gao, 2007; Ofori-Boadu et al., 2026).

Secondly, the IBCS theory captures how specific meaningful engagements and important self-attributes originate and interact to form the identity content of AEC professional identities and drive AEC-PID in AEC women undergraduates. Persistent AEC women undergraduates possess contrasting self-attributes that align well with the contrasting attributes of AEC professions (Bataille and Vough, 2020; Javaid and Pandarakalam, 2021; Ofori-Boadu et al., 2020). These contrasting self-attributes are clustered in the broader dimensions of abilities, interests, and resilience. Considering that women’s abilities are often questioned, undervalued, and rated less than men counterparts in MM STEM contexts, the possession of professional abilities is very important to women. It increases their professional credibility and self-efficacy to think, feel, and act as professionals (Wise students, 2022; Moss-Racusin et al., 2012; Cruess et al., 2014). In fact, the prove-it-again bias suggests that while women are required to demonstrate their competence, men are assumed to be competent. Therefore, women work hard, sometimes even harder than men counterparts to prove that they possess the competence required to professionals. In concurrence, Short (2019) highlighted that STEM women hold themselves to a high standard and feel the need to prove themselves beyond what is required so that they can demonstrate that they earned their position. Considering that women’s interests are often questioned and the competitive and agentic nature of MM STEM professions are perceived to be incongruent with women’s communal and altruistic goals (Su and Rounds, 2015; Eagly and Karau, 2002), the possession of professional interests is very important to women. It increases their motivation to continue to develop themselves, persist, and remain in MM STEM professional boundaries, despite challenges. Considering the complex gendered tensions that women experience in MM STEM contexts, the possession of resilience is important to women. Resilience allows women to bounce back from gendered tensions and persist in developing the abilities, interests, and networks needed to advance and integrate woman, student and professional identities. Our findings align with Fisher et al. (2026), who found that resilience in STEM post-doctoral women stems from interest, self-efficacy, and support. In fact, The Women in STEM Network (2025) emphasized that resilience is an essential skill for women to develop in MM contexts. This is because these contexts cause unique challenges and feel unwelcoming to women because they are underrepresented and undervalued in MM contexts. Yet, with the progressive resolution of gendered tensions and resilience discrepancies, women persist in MM STEM contexts long enough to explore and implement actions to transform these contexts. In agreement, The Women in STEM Network (2025) noted setbacks can transform into growth opportunities with resilient women progressing into leadership roles with a unique capacity to implement collaborative and inclusive approaches that benefit teams and build resilience in others. This aligns with the IBCS theory that highlights that PID is characterized by transitions from beneficiary to benefactress roles to change AEC contexts.

By revealing the alignment between self-attributes and the attributes of professions, the IBCS theory aligns with identity content domain theories that emphasize the importance of person-domain alignment in PID (de Moor and Olaru, 2025). AEC women undergraduates perceive AEC professions as possessing the contrasting attributes that align with their unique and multiple contrasting self-attributes. This contrasting nature was highlighted by Ofori-Boadu and Ofori-Boadu (2022) who found that 52% of first-year AEC women emphasized that contrasting mathematics and arts preferences influenced their AEC career choices. In fact, it is not uncommon for scholars to suggest that contrasts exist in education and professional domains. In their study on identity content, Sullivan and Kedrowicz (2012) noted contrasting soft and hard skills. Also, Faulkner (2000) described contrasting technical/social, abstract/concrete, and masculine/feminine attributes in engineering professions. Aligning with the IBCS theory that the blending of contrasting self-attributes may be unique to women in MM STEM contexts, Yang and Barth (2015) emphasized that while biology STEM women undergraduates tended to be only interested in people jobs, non-biology STEM women were interested equally in contrasting people and thing jobs. While Su et al. (2009) found women preferring to work with people and men preferring to work with things, the IBCS theory reveals that that AEC women undergraduates are interested in working with both things and people. Additional studies are needed to confirm if these insights explain gaps in women’s participation in STEM and MM STEM disciplines.

Furthermore, while existing studies associate broad self-attributes such as interest with AEC choices (Macdonald and Durdyev, 2021), the IBCS theory reveals the more nuanced contrasting self-attributes within the interest dimension (e.g., indoor/outdoor space interests) that aligns with the attributes of AEC professions. While stereotypes tend to restrict women to feminine professions like nursing because it is usually delivered indoors, AEC professions are more welcoming to women with contrasting indoor/outdoor interests because they can work both indoors (e.g., offices) and outdoors (e.g., construction sites). In fact, women expressed contrasting interests by wanting to work on both small and large things. Working to make small things larger drove women’s interest in constructing built environments which typically progressed from an empty lot to an occupied building or utilized infrastructure. Despite their interest in things, women undergraduates found that AEC allows them to work with people as well. They found ways to incorporate their communal values in AEC roles to include being members and leaders of student organizations. While scholars like Su et al. (2009) indicated that women did not strongly align with Holland’s (1997) realistic interests and constrained women to artistic, social/communal interests, our findings align with Ofori-Boadu et al. (2026) who found women to also possess strong realistic creative interests. We found that women who possess contrasting self-attributes which are deemed important in their professional considerations tend to express fewer and less intense personal-professional identity fit discrepancies. Therefore, they are more likely to commit to becoming AEC women professionals. We contribute to identity content literature by showing that rather than limiting women to only artistic and social interests, women can also have authentic realistic interests and technical competencies. We specify the varied, nuanced, and contrasting self-attributes that originate and develop to form AEC professional identities. Therefore, whereas workforce development models such as science, technology, engineering, arts, and math (STEAM) models promote contrasting engagements for well-rounded learning (Al-Mutawah et al., 2022), the IBCS theory postulates that STEAM engagements (Ofori-Boadu, 2018) may be critical for the origination and development of stable AEC professional identities. We extend PID literature by providing a more nuanced understanding of AEC-PID in AEC women undergraduates. Specifically, we explicate the meaningful contrasting engagements, as well as the nuanced and contrasting self-attributes that form the content of AEC professional identities in AEC women undergraduates.

Thirdly, the IBCS theory tracks and captures how meaningful engagements and important self-attributes interact and gradually evolve over time to influence varied trajectories in AEC-PID processes. We utilized four loosely connected and sequential IBCS phases to demonstrate key transition points in AEC-PID and explained how the re-specification of self-attributes shape PID trajectories. This aligns with McCall et al. (2021) who found that civil engineering undergraduates utilized iterative self-definitions to construct professional identities. While these processes are non-linear and iterative, our four IBCS phases track growth in the contrasting self-attributes within three IBCS dimensions (interest, abilities, resilience) as women transition from non-AEC novices through AEC beneficiaries to become AEC benefactresses (Waters and McAlpine, 2017). Transitions from idealistic interests in phase one to realistic interests in phase four capture increments in the desire to re-engage with solving real problems in AEC contexts and drives the motivation to surrender some personal attributes to meet AEC expectations. A similar finding was noted by Gibson et al. (2010) who found that new counselors transitioned from idealism to realism in PID. Also, Hidi and Renninger (2006) utilized four phases to capture interest development as individuals transitioned from triggered situational interest which was fleeting to well-developed interest which was independent and actively sought feedback. Also, transitions from generic abilities in phase one to professional abilities in phase four captures increments in competence and advances the professional self-efficacy needed to remain within AEC professional boundaries. Overtime, abilities and interests narrow in professional scope but increase in complexity as women re-specify these self-attributes and transition from broad AEC aspirations to align with AEC specialization aspirations. Like Martha, abilities and interests have gendered meanings and are important in PID in women because traditionally women are perceived as lacking STEM abilities and interests which make them incongruent with MM STEM professions (Eagly and Karau, 2002). Women work hard to develop abilities to prove to others that they possess the professional abilities needed to persist in AEC professions. Notably, despite their academic difficulties, women with strong interests but limited abilities still exhibit strong AEC-PID and explore ways to remain in AEC domain. This agrees with Oyserman and James (2011) who indicate that with strong professional identities, students tended to interpret difficulties as important rather than impossible to attain and therefore persist. Contrarily, women with low interests but strong abilities demonstrate weak AEC-PID and explore ways to exit AEC domains. These findings suggest that the contrasting interests dimension may be the strongest predictor of women’s PID and persistence in MM STEM programs and professions.

We found that systemic gender inequalities and gendered tensions are evident during childhood and pre-college phases. Therefore, phase one shows that the earliest career aspirations of girls were towards non-AEC professions because they lacked awareness or understanding of AEC professions. Similarly, Cheryan et al. (2017) found that girls lacked sufficient early experience and Fash and Ofori-Boadu (2025) noted that girls had misconceptions about AEC professions. Over time, gendered tensions increased in intensity as women transitioned from pre-college through undergraduate to industry contexts. Nevertheless, only a few women attribute exit intentions to gendered tensions and resilience discrepancies during early employment contexts. Rather, we found that women’s resilience strategies increase in diversity and strength as they combine new and existing negotiation mechanisms to creatively minimize discrepancies and integrate woman and professional identities. This aligns with Ross et al. (2021) who found that black women developed resilience to persist in their engineering identity development. By explaining how these IBCS dimensions evolve over time, our IBCS theory aligns with developmental social-psychological perspectives that emphasize how identities form and change over time to support professional development. We extend PID literature by providing a more holistic understanding of AEC-PID in AEC women undergraduates. Specifically, we explicate the nuanced increments and sequential links that connect evolving and contrasting self-attributes within and across phases and contexts to facilitate AEC-PID processes in women undergraduates (Crocetti et al., 2023).

Fourthly, rather than passive and homogenous followers who are restricted by systemic gender inequalities, the IBCS theory explains the diverse and iterative strategies and mechanisms utilized by undergraduate women to agentially construct their own AEC professional identities. These agentic strategies and mechanisms demand reasoning, effort, time, and resources (Afshari, 2021). Agency plays an important role in AEC-PID as progressively, women intentionally and independently explore, make meaning, assess, negotiate, and direct the re-specification of their self-attributes and subsequent engagements to align with the attributes of professions. This finding agrees with Chávez et al. (2023) who also found that subjective learning and agency were key to PID in teachers. We employ four loosely connected IBCS phases to capture how women agentially and sequentially utilize isolation, aggregation, ranking, and blending strategies to construct and align their self-attributes with progressing professional aspirations.

As shown in Figure 1, the three IBCS trajectories explicate the varied negotiation and enactment mechanisms utilized by women to resolve discrepancies, align, and integrate personal and professional identities for distinctiveness and coherence (Ma, 2023). The three trajectories capture the six different mechanisms utilized by women to resolve three levels of discrepancy intensities to achieve full (Conformers), partial (Alterers), or no (Breakers) commitment to specializations within the boundaries of the AEC professional domain. We found that with tolerable discrepancies, most conformers demonstrated agency by implementing mechanisms that facilitate commitment to specializations within their original AEC aspirations. Miller et al. (2024) noted that intra-major specializations influenced career plans in biomedical engineering undergraduates. With strong interests but lower abilities, few engineering women demonstrated constrained agency by transitioning under pressure to construction programs to conform with structural requirements. Therefore, they accepted less preferred non-original AEC options to remain within AEC professional boundaries. Yet few engineering and architecture women without ability discrepancies express agency by considering non-AEC aspirations to resolve significant or intolerable interest and resilience discrepancies. They tended to believe that their engineering skills are transferable to other disciplines. While some switched to other non-AEC programs, due to investments and need for financial stability, some plan to enter the AEC workforce upon graduation and exit later. We note there that construction women exhibit the least tendency to explore non-original AEC domains. This may be because of prior considerations of architecture and/or engineering in Phase 3.

Over time, as women resolve discrepancies, their agency grows stronger and they increase in confidence and sense of control. Therefore, they progressively transition from being more reliant on socializers like parents in phase 1 through beneficiary roles which rely on peers and experts to advancing into benefactresses roles to change contexts in phase 4. Booker et al. (2021) noted that individuals gained more control and mastery when a negative experience became positively resolved. Furthermore, individuals who maintained greater agency felt better in control of their lives, had a clearer sense of who they are, and had greater self-efficacy for life’s challenges. Therefore, over time, women actively construct their own professional identities by transitioning from isolating contrasting self-attributes towards non-AEC aspirations in phase one to blending contrasting self-attributes to integrate personal and professional attributes for congruent professional identities in phase four. In a similar study, Gibson et al. (2010) also noted how new counselors transitioned from segmentation to integration of personal and professional attributes.

While most of the mechanisms for resolving discrepancies are mostly individualistic, we found a more collectivist mechanism utilized by women to resolve resilience discrepancies. Our new *womfessionalization* concept captures a cognitive and emotional resilience mechanism utilized by women with professional self-efficacy, motivation, and sense of belonging needed to remain and resolve gendered tensions in MM STEM contexts by pursuing influential problem-solving and altruistic roles to transform innovation and inclusion for all populations. It is well-documented that MM-STEM women remain underrepresented and report higher frequencies and intensities of gendered tensions that demand more complex resilience mechanisms to develop professional identities compared to their MM STEM men counterparts and other STEM women (Cheryan et al., 2017; Hatmaker, 2013; Faulkner, 2007). However, there is no single construct to capture this more complex resilience mechanism and how it drives professional identities and commitments to transform MM-STEM contexts. Therefore, this proposed, *womfessionalization* concept provides an initial, single, and unified construct that can be utilized to gain a more nuanced and holistic understanding of how complex resilience mechanisms originate and evolve to influence how women bounce back from gendered tensions to transform MM STEM contexts.

The core conditions that enable this mechanism are: (1) Only individuals who self-identify as women can access this mechanism because being a woman is the main source of conflict that demands complex resilience mechanisms to facilitate PID in MM STEM contexts; (2) Only women engaged in MM STEM professional contexts can access this mechanism because these contexts facilitate the structural barriers and systemic inequalities that cause the gendered tensions which demand this complex resilience mechanism (Heydari et al., 2024); (3) Only women who possess professional STEM abilities and interests can access this mechanism because it is driven by the possession of professional self-efficacy and motivation that allows women to remain long enough within MM STEM contexts to bring transformation. These required professional abilities and interest requirements make *womfessionalization* significantly different from feminism because feminism does not demand professional STEM self-efficacy and motivation (Diekmann, 2015). Furthermore, in their publication of the “I am not a feminist…” article, Seron et al. (2018) explained that engineering women undergraduates reject feminism because it suggests that women do not possess the talents needed to remain in professional contexts. Therefore, they must utilize complaints and affirmative action to access professional contexts. Considering that abilities and interests are typically downgraded, women in MM STEM want to be known for being in professional domains because they meet the qualifications. While, a related feminist concept, STEMism concept aligns better with STEM women, it still draws from feminism and presents the complaint and special treatment voice which engineering women reject. Furthermore, it focuses on all STEM women and risks masking the more complex gendered tensions encountered by women in MM STEM professions. Therefore, the *womfessionalization* mechanism is significantly different from STEMinsm because it focuses on the more complex tensions faced by women in MM STEM and highlights how women possess and utilize their talents to persist and transform contexts; (4) Only women who are willing to do whatever it takes, to include integrating contrasting attributes can access this mechanism which draws from contrasting agency and communal self-attributes. This is critical because studies show women as either adopting masculine (agentic/independent) tactics by undoing gender and devaluing femaleness to gain male acceptance and networking (Ryan et al., 2020) or adopting feminine (communal/interdependent) tactics and celebrating femininity or networking with other women (Seron et al., 2018) to resolve gendered tensions and persist. Su et al. (2009) limited women to social and artistic interests, but not realistic interests. Also, Powell et al. (2009) described women as undoing femaleness to gain male acceptance. Notably, while these scholars suggest the separation of masculine and feminine attributes in PID, our findings align with a recent study by Tremmel and Wahl (2023) that suggest a shift towards the demonstration of both masculine and feminine attributes in leadership; and (5) Only women who transition from beneficiary to benefactress roles to signal strong PID are able to access this mechanism and commit to transform MM STEM contexts through innovation and inclusion for all populations. While many empirical studies tend to position women as passive responders, womfessionalization mechanisms are exhibited by women who agentially implement both individualistic and collectivist mechanisms to transform STEM contexts. The significantly higher gendered tensions experienced by women in MM STEM contexts are well documented and stakeholders continue to seek insights to improve their persistence in the STEM workforce. With this new concept, we provide a starting point to understand this unique mechanism utilized by women in these contexts to inform interventions that support PID in MM STEM contexts.

### 4.2 Research contributions

We demonstrate that the constructivist grounded theory methodology is beneficial for gaining a holistic and nuanced understanding of PID processes. It can be replicated by other scholars. This methodology yields a constructivist and longitudinal framework for investigating agency and trajectory in PID. Our model shows identity content, process, and context dimensions that are loosely linked across sequential phases as women gradually construct their own professional identities within specific contexts, while navigating gendered tensions. Our proposed, *womfessionalization,* concept provides baseline construct for gaining a more conceptually rich understanding of resilience development in women in MM STEM fields and how resilience drives commitment to transform MM STEM professions.

### 4.3 Practical contributions

Our IBCS theory informs practical applications across the three AEC disciplines and four sequential contexts in four distinct ways:

*IBCS-informed recruitment:* To accelerate girls’ awareness and understanding of the contrasting nature of AEC professions for earlier AEC decision-making, our IBCS theory facilitates the inclusion of contrasting elements in recruitment strategies (e.g., media, summer camps) (Ofori-Boadu, 2018; Ofori-Boadu et al., 2019; Hyland et al., 2022). Physical and digital strategies through educator-practitioner collaborations and media promotions can expose girls, educators, counselors, and society to contrasting aspects of AEC professions. These are particularly important during elementary and middle school stages and would reduce the time utilized to navigate the non-AEC aspirations. Outreach should extend beyond STEM to non-STEM domains such as girls with contrasting self-attributes tend to participate in contrasting engagements (Ofori-Boadu et al., 2020).
*IBCS-informed Self-Reflection:* To enhance agency in AEC-PID, our IBCS theory facilitates the inclusion of contrasting elements in the development of self-reflection tools (e.g., journaling, prompts). This will accelerate self-awareness and the understanding of AEC-relevant contrasting interests, abilities, resilience, and engagements to guide career choices in multi-level contexts. Such tools can foster the early identification and resolution of discrepancies. Therefore, they should be embedded in personal, educational, extracurricular and employment engagements to develop realistic expectations and minimize misconceptions (Ran et al., 2023). Resources should also highlight nuanced differences among AEC disciplines and specializations. Counselors, advisors, educators, policy makers and other stakeholders should be trained to support self-reflection and self-regulation skill development in women.
*IBCS-informed education:* To construct the contrasting self-attributes needed for AEC-PID in educational environments, our IBCS theory informs the development and implementation of contrasting visual-kinesthetic STEAM and AEC engagements (Stone, 2022) in indoor and outdoor environments with women and men socializers. Early introduction to AEC specializations and other interdisciplinary offerings such as minors and certificates can resolve discrepancies early. Furthermore, we recommend an increase in AEC expert content in gateway experiences and access to well-designed beneficiary and benefactress engagements. It is imperative that benefactress engagement (e.g., leadership) opportunities are provided for women to engage the *womfessionalization* mechanism. To build resilience, we encourage early participation in co-ed sports and more inclusive cultures in high school engineering courses. Women-exclusive organizations must be designed as temporary incubators that facilitate transitions into gendered AEC domains (Shane et al., 2012). Furthermore, there should be an increase in the technical and professional engagements in women-exclusive organizations. Educators, administrators, and policy makers should be trained to provide supportive systems such as employing women professors.
*IBCS-informed employment:* To develop and utilize contrasting self-attributes in industrial environments, our IBCS theory informs the design and implementation of contrasting developmental strategies in early employment contexts (Carrasco and Lopez, 2025). Tailored orientations can involve both women and men mentors. Employment rotations can expose women to AEC specializations and options within an organization, as well as peripheral roles beyond organizational boundaries (e.g., consultant, client). Considering early career women, particularly engineering women, may have long-term plans to exit AEC professions, we recommend customized career guidance that will quickly detect discrepancies and tailor support. Employees, particularly direct supervisors, should be trained to support women.

## 5 Limitations

We assumed that RPs were objective and truthful. Also, their childhood narrations drew from accurate memories while future aspirations narrations were based on rational speculations. Findings are limited to women who self-identify as undergraduate AEC women in the U. S and are concerned about inequality issues. They tended to self-select and participate in this research study. Due to the constructivist grounded theory methodology adopted for this study, findings are dependent on subjective interpretations made by the researchers. Considering that research participants were from U.S. institutions, generalizing findings beyond the U.S. should be done with caution.

## 6 Conclusion

Majority of research on PID in women in masculine and male-dominated STEM professions utilize cross-sectional designs that highlight structural barriers at a single point in time and appear to position women as a passive and homogenous group responding to gendered threats. The originality of our work lies in our adoption of a constructivist and longitudinal grounded theory approach for gaining a more nuanced and holistic understanding of agency and trajectories in PID in AEC women undergraduates, who are women professionals in training and must navigate gendered tensions in MM AEC contexts. Therefore, we develop a novel IBCS grounded theory that captures the range of self-attributes and engagements that interact and evolve to influence the diverse negotiation mechanisms and enactments that women utilize to agentially construct their own sense of becoming AEC women professionals. Utilizing four phases and three dimensions, we propose a framework that loosely links identity content and process dimensions that show how participation in increasingly complex, realistic, and gendered engagements provide women with the opportunities to agentially originate, re-specify, and blend their nuanced contrasting self-attributes to align with the inherent contrasting attributes of AEC professions. Notably, agency increased over time but varied across women undergraduates due to individual differences. While identity-affirming engagements enhance PID, identity-conflicting engagements generate discrepancies when women struggle to blend contrasting self-attributes. Discrepancies were mostly attributed to society’s limited understanding of the nuanced differences among AEC disciplines and specializations and systemic gendered inequalities which facilitate gendered tensions and inaccurate expectations. Due to varied meanings, our study revealed varied negotiation mechanisms and enactments utilized by women to resolve these discrepancies and transition from beneficiary to benefactress roles. This transition marks a significant milestone in PID and suggests that benefactress roles are needed in undergraduate programs to facilitate AEC-PID in women undergraduates. Nevertheless, discrepancies persist and some women resolve tolerable discrepancies through refinement, reformulation, and *womfessionalization* mechanisms. These mechanisms allow women to pursue AEC specializations and disciplinary options that facilitate the optimized integration of personal and professional identities. Our new, *womfessionalization,* concept expands PID literature by capturing a unique resilience mechanism by which women bounce back from gendered tensions and gain confidence to utilize problem-solving and altruistic strengths to transform innovation and inclusion in MM STEM contexts. Notably, women with significant discrepancies exhibit sub-optimal integration of personal and professional identities in AEC-PID while women with intolerable discrepancies adopt the withdrawal mechanism and switch to non-AEC aspirations.

While existing undergraduate AEC systems have supported AEC-PID in women, we recommend IBCS-informed recruitment/outreach strategies, self-reflection, education and employment policies and strategies to better support agency in women and provide opportunities to advance in their thinking, feeling, and acting as AEC women professionals. Insights inform phased and targeted interventions and investigations to continue dialogues for enhancing agency to increase women participation and reducing AEC workforce shortages.

### 6.1 Future studies

(1) Quantitative studies will validate aspects of the IBCS theory which holds potential to inform theory, research, and practical applications across AEC disciplines and multi-level educational contexts; (2) Understanding the extent to which the IBCS theory applies to undergraduate AEC men and women in other male-dominated STEM disciplines may extend its applicability; (3) Understanding how women make meanings of microprocesses within specific engagements will inform best practices; (4) The role that specializations play in AEC-PID should be studied; (5) Disciplinary differences in AEC-PID should be investigated; and (6) Studies are needed to increase understanding of the linkages among the more nuanced elements and microprocesses within our proposed *womfessionalization* dimension.

## Data Availability

The datasets presented in this article are not readily available because the data used during this study is confidential in nature and may only be provided with restrictions. Due to the sensitive nature of the interview questions and the need to protect the privacy of research participants, they engaged in this research with the understanding that only this research team would have access to their video recordings. Therefore, there will be no public sharing of data. Both privacy and confidentiality in this research are protected by our Institutional Review Board. However, upon reasonable requests from interested researchers, the corresponding author, AO-B, may share summarized and/or anonymized data after ongoing analysis is completed. Requests to access the datasets should be directed to AO-B, andreao@ncat.edu.
